# The ATXN2 Orthologs *CID3* and *CID4*, Act Redundantly to In-Fluence Developmental Pathways throughout the Life Cycle of *Arabidopsis thaliana*

**DOI:** 10.3390/ijms22063068

**Published:** 2021-03-17

**Authors:** Zaira M. López-Juárez, Laura Aguilar-Henonin, Plinio Guzmán

**Affiliations:** Center for Research and Advanced Studies of the IPN, Irapuato Unit, Genetic Engineering Department, Irapuato 36824, Mexico; zaira.lopez@cinvestav.mx (Z.M.L.-J.); laura.aguilar@cinvestav.mx (L.A.-H.)

**Keywords:** flowering-time, leaf development, leaf senescence, miR169

## Abstract

RNA-binding proteins (RBPs) are key elements involved in post-transcriptional regulation. Ataxin-2 (ATXN2) is an evolutionarily conserved RBP protein, whose function has been studied in several model organisms, from *Saccharomyces cerevisiae* to the *Homo sapiens*. ATXN2 interacts with poly(A) binding proteins (PABP) and binds to specific sequences at the 3′UTR of target mRNAs to stabilize them. *CTC-Interacting Domain3* (*CID3*) and *CID4* are two ATXN2 orthologs present in plant genomes whose function is unknown. In the present study, phenotypical and transcriptome profiling were used to examine the role of *CID3* and *CID4* in *Arabidopsis thaliana*. We found that they act redundantly to influence pathways throughout the life cycle. cid3cid4 double mutant showed a delay in flowering time and a reduced rosette size. Transcriptome profiling revealed that key factors that promote floral transition and floral meristem identity were downregulated in *cid3cid4* whereas the flowering repressor *FLOWERING LOCUS C* (*FLC*) was upregulated. Expression of key factors in the photoperiodic regulation of flowering and circadian clock pathways, were also altered in *cid3cid4*, as well as the expression of several transcription factors and miRNAs encoding genes involved in leaf growth dynamics. These findings reveal that *ATXN2* orthologs may have a role in developmental pathways throughout the life cycle of plants.

## 1. Introduction

RNA-binding proteins (RBPs) mediate a vast array of post-transcriptional processes by interacting with target RNAs. RBPs exert conserved functions among eukaryotic organisms, acting in splicing, polyadenylation, translation, stability, editing, and mRNA localization. RBPs are highly represented in the genome, and there are at least 15,000 in humans and 1800 in plants. They comprise diverse classes that include many conserved domains that mediate RNA interaction [1,2].

A well-conserved class of RBPs across eukaryotic organisms is the poly(A) binding protein (PABP). They participate in various central activities in the cell from mRNA assembly, transport, and decay, to translation initiation; PABP bridge the 3′ mRNA tail to the 5′ translation complex eIF4F through eIF4G. A canonical PABP protein contains at the amino-terminal region four copies of the RNA recognition motif (RRM), and at the carboxy-terminal, the PABPC domain, also known as MLLE or CTC. The MLLE domain binds the 12 amino acids long domain named PABP interacting motif 2 (PAM2) present in various regulatory proteins [3,4,5].

Ataxin-2 (ATXN2) is a protein conserved across eukaryotic organisms, that in humans is a genetic determinant of at least two neurodegenerative diseases, spinocerebellar ataxia type II (SCA2) and the amyotrophic lateral sclerosis (ALS) [6,7]. The conserved domains of ATXN2 include a PAM2 domain that interacts with PABP, a Like RNA splicing domain Sm1 and Sm2 (LSm) that binds RNA, and a Like-Sm-associated domain (LSmAD) containing a trans-Golgi signal [8]. Besides *Homo sapiens*, ATXN2 orthologs have been studied in the model organisms as *Saccharomyces cerevisiae*, *Caenorhabditis elegans*, *Drosophila melanogaster*, and *Mus musculus*. Roles concerning basic RNA functions, in the regulation of cytoplasmatic untranslated messenger ribonucleoproteins (mRNPs) granules and in the target of rapamycin (TOR) protein kinase signaling are ubiquitous to the model organisms, suggesting similar mechanisms behind the function of ATXN2 orthologs in eukaryotes [8,9,10,11]. A central molecular mechanism of Ataxin-2 is to bind the 3′UTR of mRNAs. The yeast ATXN2 ortholog Poly(A)-binding protein-binding protein 1 (Pbp1) was formerly inferred to have a role in polyadenylation by binding to the poly(A) tails of mRNAs [12]. More recently, mammalian ATXN2 was found to bind to the U-rich element AUUU(U/A) located at the 3′ UTR of target mRNAs through LSm domain. This binding improves the stability of target mRNAs and consequently increases levels of target proteins [13]. Ataxin-2 also interacts with POLY(A) POLYMERASE (PAP)D4, a noncanonical PAP, resulting in an extended poly(A) tail of specific mRNAs and a consequent improvement of translation and the stability of target mRNAs [14].

The mRNPs accumulate in the cytoplasm in processing-bodies (P-bodies) that consist of untranslated mRNAs and the machinery of mRNA decay and translation repression, as well as in stress granules that consist of untranslated mRNAs, translation initiation factors, and RNA binding proteins [15]. Neuronal RNP granules are similar granules that consist of untranslated mRNAs and regulatory proteins that support their movement to axons or dendrites [16]. *Saccharomyces cerevisiae*, *Drosophila*, *Caenorhabditis elegans*, and human ATXN2 orthologs interact with the RNA helicase DDX6, a critical factor found in P-bodies that is also found in stress granules. DDX6 is regarded as a component of protein complexes that direct post-transcriptional regulatory activities [17]. Pbp1 functions in stress granules assembly in response to heat shock or glucose deprivation and overexpression of Pbp1 and Dhh1 (the yeast DDX6 ortholog) inhibits cell growth [18]. Atx-2, the *C. elegans* ATXN2 ortholog, operates in the translational regulation of specific mRNAs in the germ-line cell and interacts with other regulatory RNA binding proteins; it has also been established that Atx2 also regulates stress granules and P-bodies assembly [19]. Me31B, the Drosophila DDX6 ortholog, is a basic component of the ATX2 complex to maintain the circadian clock [11]. Moreover, mRNP assemblies have an impact on the long-term neuronal plasticity and memory formation [20]. RCK/p54, the DDX6 ortholog in humans, interacts with ATXN2 disturbing P-bodies and stress granules assembly in a concentration-dependent manner [21]. mRNP granules are dependent on ataxin-2 intrinsically disordered regions (IDRs) for formation [22]. IDRs are polypeptide segments devoid of a unique three-dimensional structure under physiological conditions that assume a distinct conformation after interacting with a macromolecular partner [23]. ATXN2 contains a middle IDR (mIDR) and a carboxy-terminal IDR (cIDR), and the presence of both IDRs is required for granules formation [20].

The TOR protein kinase is an element of a fundamental pathway that regulates cell growth, development and metabolic homeostasis, and impacts aging and life span. TOR assembles into the multiprotein complexes TOR complex 1 (TORC1) and TORC2 [24]. In yeast, Pbp1 is a regulator of TORC1, a serine/threonine kinase that functions as a main cell growth regulator in response to many and diverse signals, such as stress conditions, development, and nutrients; Pbp1 controls TORC1 activity after sensing cellular redox state [25,26]. In *C. elegans*, Atx2 regulates cell and body size, as well as fat content through the mTOR pathway [27]. In mammals, ATXN2 is induced upon nutritional stress, and modulates mRNA translation through the mTOR pathway interacting with preinitiation complex components [28].

The interaction mediated by PAM2 and MLLE is conserved in the plant kingdom. Four distinct classes of MLLE-interacting proteins designated as CTC-interacting domains (CIDs) have also been identified in the model plant *Arabidopsis thaliana*. Three of them are likely RBPs since they contain known RNA-binding domains [29,30]. CID A includes two members in *A. thaliana*, *CID1*, and *CID2*, encoding putative transcription-activating domains. *CID1* is involved in early stress responses and is also known as the *EARLY RESPONSIVE TO DEHYDRATION* 15 (*ERD15*) gene [31]; *CID2* is an uncharacterized gene [30]. CID C class contains *CID5*, *CID6*, *CID7*, encoding the Coupling of Ubiquitin conjugation to ER degradation (CUE) domain. *CID5* and *CID6* are highly related on sequence; *CID5* is also known as the *INCREASED POLYPLOIDY LEVEL IN DARKNESS 1-1D* (*IPD1-1D*) gene, with a role in the regulation of endoreplication cell cycles [32]. CID7 also bears the RNA binding domain Small MutSRelated (SMR) [30]. Class D includes *CID8*, *CID9*, *CID10*, *CID11*, *CID12*, and *CID13* that encode two RNA recognition motifs; the function of Class D members is unknown [30].

Class B comprises four *ATXN2* orthologs: *CID3*, *CID4*, *CID16*, and *CID17*. CID3 and CID4 contain three canonical ATXN2 domains, PAM2, LSm and LSmAD, whereas, in CID16 and CID17, the LsmAD domain is absent [33]. The purpose of the present research was to initiate the functional analysis of the ATXN2 orthologs in *A. thaliana*. We found that single *cid3* and *cid4* mutants did not display any readily observable phenotype, whereas double *cid3cid4* showed a delay in flowering time. Transcriptome profiling at bolting indicates that the expression of several well-characterized flowering time genes and flower identity genes was affected. Genes involved in leaf dynamics were also affected, suggesting that *ATXN2* orthologs in *A. thaliana* may function during developmental processes.

## 2. Results

### 2.1. CID3 and CID4 Act Redundantly to Modulate Rosette Size and Flowering Time

**Figure 1:**
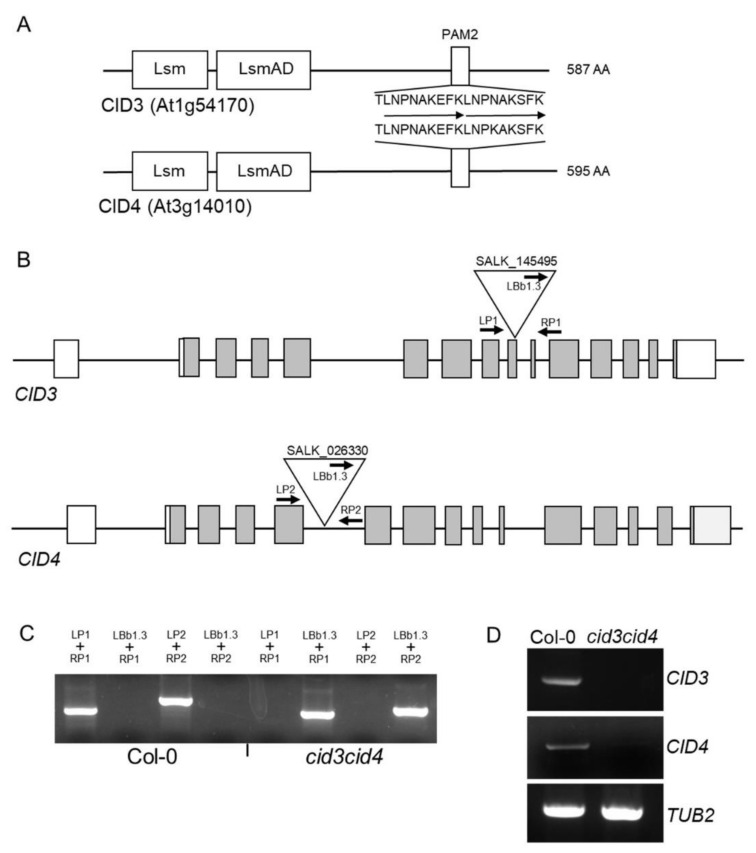
Generation of the double *cid3cid4* mutant. (**A**) Schematic presentation of protein models for CID3 and CID4. Position of Lsm, LsmAD, and PAM2 is display. The PAM2 sequence is identical, showing the tandem reiteration of the PAM2 sequence found in these two proteins. (**B**) Schematic presentation of gene models for *CID3* and *CID4*. Exons are represented by rectangles and introns by lines; gray rectangles denote coding sequences. Triangles point location of the SALK T-DNA insertions in *CID3* and *CID4*. Arrowheads indicate the position and orientation of the oligonucleotide primers used in genotyping, LBb1.3 is specific to the left border of the T-DNA, LP1, and RP1 are gene specific primers for each of the two genes. (**C**) PCR amplification products obtained from genomic DNA extracted from the wild type Col-0 and the *cid3cid4* double mutant were resolved in 1.5% (*w/v*) agarose gels; they confirm the homozygous T-DNA insertion at both loci. Amplifications products were only obtained in Col-0 DNA (*CID3*, 430bp, and *CID4*, 580bp), and T-DNA products only on *cid3cid4* DNA. (**D**) Expression analysis of *CID3* and *CID4* by RT-PCR in wild-type and *cid3cid4* mutant lines. Amplification products were fractionated into a 1.5% (*w*/*v*) agarose gels; tubulin TUB2 genes was used as constitutive control.

*CID3* and *CID4* encode the archetypal domains found in ATXN2 proteins (Figure 1A). To begin assessing the function of the ATXN2 orthologs in *A. thaliana*, we inspected the effect of loss of function mutations in the *CID3* and *CID4* genes. The T-DNA insertion mutants SALK_145495 disrupted at the 9th exon of *CID3*, and the SALK_026330 disrupted at the 5th intron of *CID4* were used (Figure 1B) [34].

**Figure 2:**
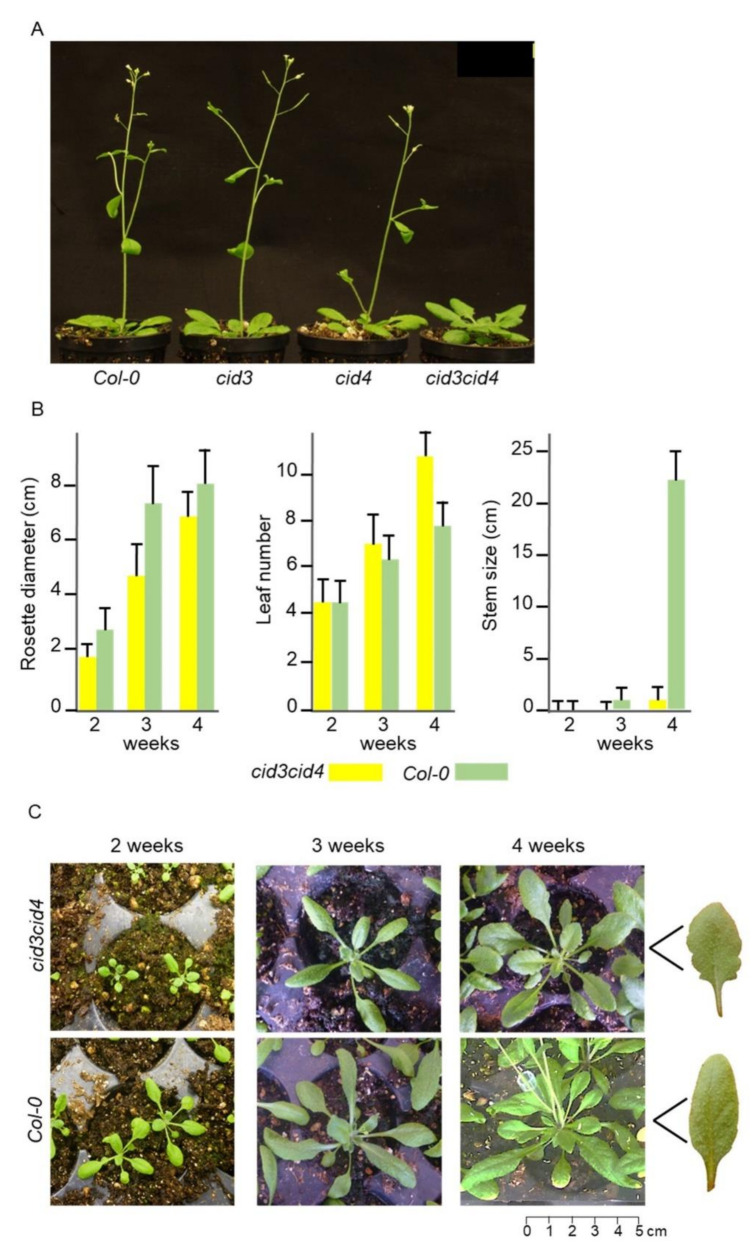
Comparison of the Col-0 and *cid3cid4* lines. (**A**) Flowering time phenotype, a delay in flowering occurs in the double *cid3cid4* mutant. (**B**) Comparison of rosette diameter, number of leaves, and stem size during growth on standard condition; mean values of 20 plants are displayed. (**C**) Comparison of rosettes of the experiment in (**B**) is displayed; a 5cm scale is shown. Representative rosette leaves from adult *cid3cid4* and Col-0 plants are shown to the right of the 4 weeks panel. Plants were grown as described in Materials and Methods.

Homozygous lines for the T-DNA insertions were indistinguishable from wild-type Col-0; no major macroscopic phenotypic defects were detected at seedling, young and adult stages (Figure 2A). Then, we crossed *cid3* and *cid4* single mutants to obtain the double homozygous; full-length transcripts were not detected by RT-PCR in *cid3cid4* lines (Figure 1C,D). In the *cid3cid4* double mutant, the days to bolt were delayed by about 10 days, suggesting a defect in flowering time (Figure 2A). Consistently, the number of rosette leaves at bolting increased in the double mutant (Figure 2B). *cid3cid4* also showed reduced rosette size, which was more prominent through 2–3 weeks-old plants than plants through the transition to the adult vegetative stage (35% and 15% reduction, respectively), and an increase in leaf margin serration (Figure 2B,C). These observations suggest that the ATXN2 orthologs affect the vegetative growth as well as flowering time in *A. thaliana*.

### 2.2. Transcriptomic Alterations Exhibited in cid3cid4

**Figure 3:**
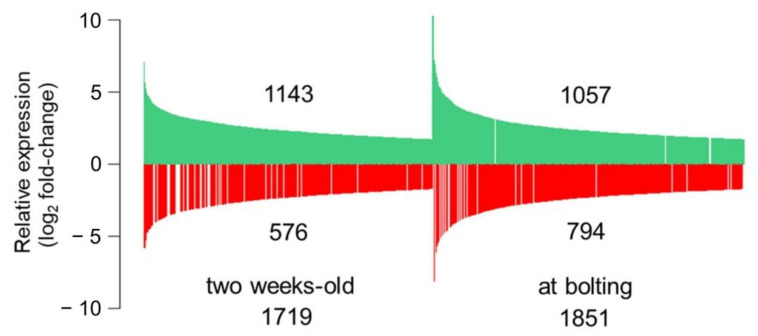
Influence of *CID3* and *CID4* on the transcriptome. The number of differentially expressed genes between the *cid3cid4* mutant and wild-type (*A. thaliana* Col-0) 2 weeks-old plants (left panel), and at bolting (right panel). Individual genes are represented by a bar. Bar length corresponds to the relative expression, upregulated genes in green, and downregulated genes in red.

An experiment was designed to assess the impact of CID3 and CID4 during vegetative growth to reproductive transition by contrasting the transcriptome of the double *cid3cid4* mutant with wild type Col-0 plants, using microarray analysis. Two biological duplicates were prepared from groups of plants grown under two conditions: from 2 weeks-old plants and plants at bolting (see Material and Methods; Figure 3). Principal component analyses (PCA) validated that the *cid3cid4* and Col-0 were different and that the data from the replicas were related (Supplementary Figure S1). Differentially expressed genes were detected using an ArrayXS Arabidopsis microarray containing 30,541 genes. Fold values were estimated on log2-normalized between the duplicates, with an absolute log2 fold-change cutoff ≥1.7 (see Material and Methods). The number of genes regulated in 2 weeks-old plants and at bolting was similar, suggesting an equivalent effect of CID3CID4 on gene expression at both stages. On the other hand, the number of upregulated genes in *cid3cid4* was greater than the number of downregulated genes under both conditions. Next, Gene Ontology (GO) classification was performed using the PANTHER Classification System with the Protein Class, Biological Process and Molecular Functions (Supplementary Figure S2). The five most significant GO terms were common between the differential expressed gene at both stages, weeks-old and at bolting, for the Protein Class (interconversion enzyme, gene-specific transcriptional regulator, transporter, transmembrane signal receptor, and protein modifying enzyme) and the Molecular Function (catalytic activity, binding, molecular function regulator, transporter activity, and molecular transducer activity) lists. For Biological Process, three GO terms were common (cellular process, biological regulation, and response to stimulus). This broad analysis alludes to an enrichment of regulatory terms from the lists of differential expressed genes.

### 2.3. The Transcriptomic Analysis Exposes Gene Expression Alteration during Young to Reproductive Transition in cid3cid4

**Figure 4:**
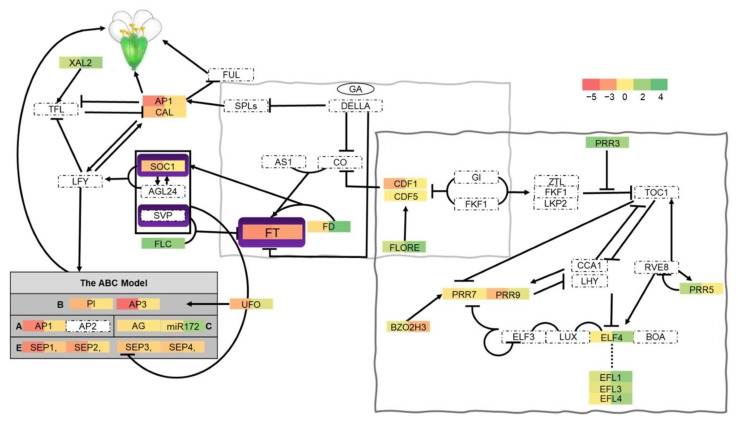
Schematic representation of gene networks regulating flowering time showing differentially expressed genes in *cid3cid4*. Gene regulatory networks are based on previously reported work [36,37,38,39,40,41]. Black arrows and blunt head arrows indicate positive or negative effects, respectively, in the pathway. Differentially expressed genes between the *cid3cid4* mutant and wild-type Col-0 are displayed in rectangles divided in two zones, each one of them in a green–yellow–red scale, upregulated genes are highlighted in green shades, and downregulated genes are highlighted in red shades. The left zone includes the values of 2 weeks-old plants, and the right zone the plants at bolting. Genes within the dotted rectangles did not show differential expression. Floral integrator genes are shadowed in violet. Floral meristem identity genes of the ABC model are shadowed in gray. A light gray rectangle encircles genes involved in photoperiodic flowering, and a gray rectangle encircles genes involved in the circadian clock. The dotted line below *ELF4* connects to three *ELF4* paralogs, *ELF4-like1,3* and *4* whose function has not been well established. Gene code values of the differentially expressed genes are displayed in Supplementary Table S1.

The transitions from young to adult and reproductive stages have been extensively worked out over the last decades, and many components of such complex and multifaceted pathways have been characterized at the expression level. Complex developmental and environmental signaling networks control flowering time [35,36,37,38]. The most noticeable phenotypic changes in the *cid3cid4* double mutant are the 10-day delay in flowering-time and the reduced rosette size, suggesting possible alterations during the vegetative to flowering transition. Therefore, we first inspected the key genes involved in flowering activation. Flowering time is finely regulated by several networks that converge on the floral integrator genes *FLOWERING LOCUS T* (*FT*), a phosphatidylethanolamine-binding protein component of florigen, and the MADS-box transcription factor *SUPPRESSOR OF OVEREXPRESSION OF CONSTANS* (*SOC1)* [39]. *FT* was markedly downregulated in *cid3cid4* both in 2 weeks-old plants and at bolting (log2FC = −3.41 and −2.75, respectively), whereas *SOC1* was downregulated in 2 weeks-old plants to a minor extent (log2FC = −1.60) (Figure 4). Expression of *CONSTANS* (*CO*), a zinc-finger transcription factor that acts on these two flowering time integrators, was not affected in *cid3cid4*. Then, we inspected the expression of *FLOWERING LOCUS C* (*FLC*), a key MADS-box transcription factor involved in flowering repression; FLC inhibits transcription of *FT* and *SOC1* [35]. *FLC* expression was upregulated in *cid3cid4* both in 2 weeks-old plants and at bolting (Figure 4). Thus, based on the expression of these regulators, flowering activation was downregulated in the *cid3cid4* mutant, whereas flowering repression was upregulated.

FT and SOC1 promote floral transition by triggering the expression of two central transcription factors specifying floral meristems and floral patterning, the MADS-box APETALA1 (AP1) and the helix-turn-helix LEAFY (LFY) [42]. In concert, FT interacts with the bZIP transcription factor FD [43], whose expression, as well as that of AP1, was downregulated in the juvenile stage, whereas LFY expression was not affected in *cid3cid4* (Figure 4). In addition, CAULIFLOWER (CAL), an AP1 paralog that is partially redundant, was also downregulated in 2 weeks-old plants in *cid3cid4*. AP1 is a transcription factor that activates the expression of the floral homeotic genes, the MADS-box transcription factors APETALA3 (AP3) and PISTILLATA (PI), to define organ identity (petals and stamens) [44]. Both AP3 and PI were downregulated in 2 weeks-old plants in *cid3cid4* (Figure 4). Similarly, *UNUSUAL FLORAL ORGANS* (*UFO*), an F-box gene that may assemble a ubiquitin ligase required for AP3 and PI function [45], was also downregulated in 2 weeks-old plants in *cid3cid4*. miR172 is another important element for floral identity, restricting AP2 activity [46]; miR172 expression was found upregulated in *cid3cid4* at bolting. Four additional MADS-box transcription factors, SEPALLATA1/2/3/4 (SEP1/2/3/4), which are functionally redundant, promote floral meristem identity in all floral organs [47]. The four SEP genes were downregulated in *cid3cid4* in 2 weeks-old plants, showing a greater effect was observed on SEP1/2 (log2FC = −3.73 and −2.82, respectively) than on SEP3/4 (log2FC = −1.56 and −1.49, respectively) (Figure 4). Consequently, key transcription factors driving floral identity were downregulated in the *cid3cid4* mutant.

Then, we examined genes from major pathways that influence the reproductive transition, and we found altered expression in key factors of the photoperiodic flowering and circadian clock pathways [48]. The photoperiod pathway has a major influence on flowering time, integrating seasonal information, and adjusting development and reproduction to environmental changes [49]. FT is the main output of this pathway, a mobile signal transported from the leaf to the shoot apex to initiate flowering. Several factors are essential to integrate the timing and location of FT expression. Among them, CYCLING DOF FACTOR (CDF) transcription factors are transcriptional repressors whose expression oscillates under different day-length conditions, setting up daily expression patterns. CDF redundantly works to repress the expression of FT and CO by binding to their promoters [50]. The expression of two CDFs was likely to be impaired in *cid3cid4* (Figure 4, enclosed in a light gray rectangle). *CDF1* was downregulated in 2 weeks-old plants, and although expression of CDF5 was not altered, a long noncoding RNA, FLORE, was upregulated in *cid3cid4*, mostly at bolting. FLORE is a natural antisense transcript of CDF5, which enhances flowering by repressing CDF5 as well as other CDFs [40].

PSEUDO-RESPONSE REGULATORS (PRRs), PRR3, PRR5, PRR7, and PRR9, are important circadian clock elements. They regulate the expression of CCA1 (CIRCADIAN CLOCK-ASSOCIATED 1) and LHY (LATE ELONGATED HYPOCOTYL), the core components of the circadian oscillator system, each one of them exhibiting a distinct temporal expression pattern throughout the day. Expression of *PRR9* is highest in the morning, then PRR7 and PRR5 are sequentially expressed about 8 h after dawn, and subsequently, PRR3 expression peaks in the evening. A complex transcriptional network functioning in a negative and positive fashion between PRRs and CCA/LHY has been proposed [51,52,53]. PRR3 and PRR5 were both upregulated in *cid3cid4* in 2 weeks-old plants and PPR3 at bolting as well. While the expression of PRR7 was not altered in *cid3cid4*, expression of bZIP transcription factor bZIP63, which is required for the proper oscillation of PRR7, was downregulated at bolting (Figure 4, enclosed in a gray rectangle) [41]. Conversely, PRR9 was downregulated in 2 weeks-old plants in *cid3cid4*.

EARLY FLOWERING 4 (ELF4) is another component of the circadian clock that, together with ELF3 and LUX, is essential to maintain the evening clock rhythmicity [54]. ELF4 expression was upregulated in *cid3cid4* at bolting. Four *ELF4* paralogs have been identified in *A. thaliana*, ELF4-like1 to 4 [55], and the expression of two of them (*EFL1* and *EFL3*) was also upregulated in *cid3cid4* at bolting (Figure 4, connected by a pointed line in). The detailed function of these paralogs is unknown; nevertheless, initial observations suggest that *EFL1* and *EFL3* are involved in flowering time regulation [55].

### 2.4. Influence of CID3CID4 on Leaf Growth Dynamics Transcriptome

**Figure 5:**
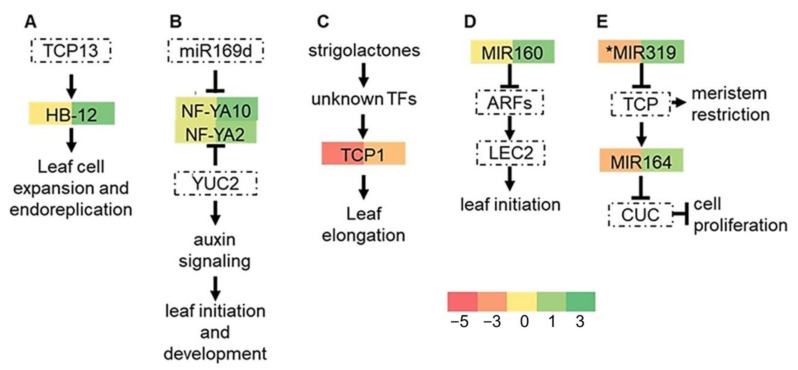
Schematic representation of gene networks regulating leaf dynamics showing differentially expressed genes in *cid3cid4*. Gene regulatory networks regulating leaf development are based on previously reported work: (**A**) [57], (**B**) [58], (**C**) [59], (**D**) [60], (**E**) [61,62]. Differential expressed values are presented as described in Figure 4. Gene code values of the differentially expressed genes are displayed in Supplementary Table S1.

The *cid3cid4* double mutant displaying a reduced rosette size phenotype and an increase in leaf margin serration suggests an alteration in leaf growth dynamics. The diverse phases of leaf growth and development are modulated by several gene networks that comprise transcription factors and microRNAs (miRNAs) [56]. To uncover such genes whose expression was altered in *cid3cid4*, we look for differentially expressed genes coding key transcription factors and microRNA precursors. We found that expression of Homeodomain leucine zipper (HB) HB-12 transcription factor was upregulated in *cid3cid4* at bolting (Figure 5A). Likewise, expression of two Nuclear Factor Y genes (NF-Y) NF-YA2 and NF-YA10 was upregulated in *cid3cid4* at bolting (Figure 5B). HB-12 regulates leaf growth during cell expansion, and NF-YA2 and NF-YA10 through auxin-mediated leaf growth [57,58]. Expression of the TEOSINTE BRANCHED1, CYCLOIDEA, and PCF (TCP) TCP1 transcription factor was downregulated in 2 weeks-old plants in *cid3cid4* (Figure 5C); it regulates leaf development via the phytohormone strigolactone signaling [59]. Expression of pre-miR160c was upregulated in *cid3cid4* at bolting (Figure 5D). miRNA160c participates during the initiation of leaf development, regulating auxin response factors [60]. miR164 and miR319 modulate leaf morphology [61,62]; pre-miR164b was downregulated in 2 weeks-old plants in *cid3cid4*, and pre-miR319 was upregulated at bolting (Figure 5E).

NO APICAL MERISTEM (NAM), ATAF1/2, CUP-SHAPED COTYLEDON2 (CUC2) (NAC), and WRKY (encodes the conserved sequence WRKYGQK) are two large families of plant-specific transcription factors with several of their members regulating the onset and progression of leaf senescence, the final stage of leaf development [63]. In *A. thaliana*, the NAC and WRKY families comprises 117 and 74 members, respectively [64,65]. Statistical analysis showed that the number of differentially expressed NAC and WRKY transcription factors was indeed over-represented (see Materials and Methods). Expression of 10 NAC and eight WRKY genes known to be involved in leaf senescence was differentially regulated in *cid3cid4*. All NAC transcription factors were upregulated, four of them in 2 weeks-old plants (JUB1, NAC016, NAC087, NAC090) and seven of them at bolting (ORS1, ORE1, JUB1, NAC029, NAC072, NAC019, NAC055); JUB1 was upregulated at both experimental stages.

**Figure 6:**
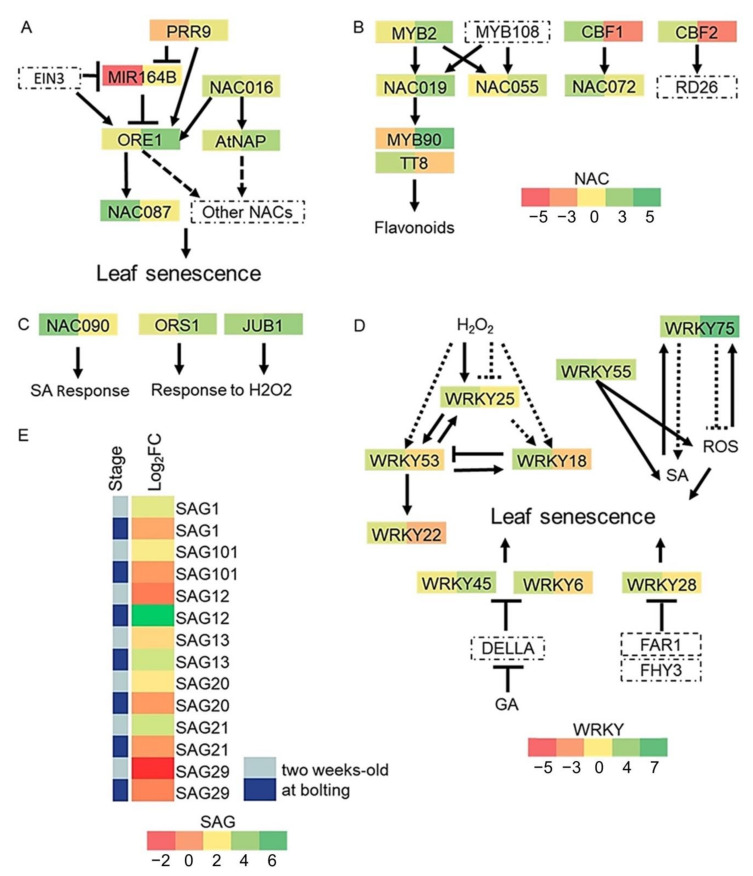
Schematic representation of gene networks regulating leaf dynamics showing differentially expressed genes in *cid3cid4*. Networks of NAC transcription factors and WRKYs based on previously reported work (**A**) [66,67], (**B**) [67], (**C**) [68,69,70], (**D**) [63,71,72,73,74,75,76,77,78]. NAC scale includes (**A**), (**B**), and (**C**). (**E**) Heat map displaying differentially expressed SAG genes in *cid3cid4*. Pale blue color corresponds to 2 weeks-old plants and dark blue color to plants at bolting. Differential expressed values are presented as described in Figure 4. Noncontinuous lines represent nondirect interactions. Gene code values of the differentially expressed genes are displayed in Supplementary Table S1.

Gene regulatory networks that connect NAC gene expression and interaction data have been predicted in *A*. *thaliana*. NACs can act as positive and negative regulators. To control leaf senescence, miR164 negatively regulates ORE, and the circadian clock component PRR9 suppresses and transcriptionally activates this module, respectively [66]. EIN3, a downstream transcription factor of EIN2 in the ethylene signaling cascade, activates ORE1 and NAC029/AtNAP, which are also likely to be direct targets of NAC016. ORE1 and NAC029/AtNAP regulate the expression of several NACs expression, NAC087 among them (Figure 6A) [67]. Another complex regulatory network is based on the phylogenetically related NACs: ANAC019, ANAC055, and NAC072/RD26. Transcription factors that activate their expression have been identified. The MYB transcription factors MYB2 and MYB108 regulate expression of NAC019 and NAC055, while CBF transcription factors CBF1 and CBF2, regulate that of NAC072/RD26 [67]. MYB2, CBF1 and CBF2 were differentially regulated in *cid3cid4*, as well as, MYB90 and TT8, downstream targets of NAC019; MYB90 and TT8 are transcription factors that activate flavonoid biosynthesis (Figure 6B) [67]. Other NACs with a role in senescence that were differentially regulated in *cid3cid4* were NAC090, which regulates the salicylic acid response, and ORS1 and JUB1 involved in response to hydrogen peroxide (Figure 6C) [68,69,70].

Six WRKY transcription factors were upregulated in 2 weeks-old plants (WRKY6, WRKY18, WRKY25, WRKY28, WRKY55, WRKY75) and four at bolting (WRKY45, WRKY53, WRKY55, WRKY75); WRKY55 and WRKY75 were upregulated in both stages, while WRKY22 was downregulated at bolting. Gene regulatory networks connect various WRKY transcription factors (Figure 6D). WRKY53 is a main component of a network that controls early senescence that is regulated by hydrogen peroxide [71]. It is regulated by various mechanisms and connects senescence with stress responses. WRKY18 and WRKY25 function upstream WRKY53, and feedback regulation is exerted among them [72]. WRKY22 has also been described as a target of WRKY53, having a role in dark-induced senescence [73]. WRKY55 and WRKY75 regulate leaf senescence by controlling the accumulation of salicylic acid (SA) and oxygen species (ROS), which are well-known inducers of leaf senescence [74,75]. WRKY6 and WRKY45 interact with DELLA proteins, components that mediate gibberellin signaling to regulate senescence [76,77]. WRKY28 regulates light-mediated leaf senescence [78].

Sets of senescence-associated genes (SAG) have been identified with enhanced expression during senescence, and they are commonly used as markers of plant senescence [79,80]. Seven SAG genes were recovered as differentially expressed genes in *cid3cid4*. Six were upregulated in *cid3cid4*, five in 2 weeks-old plants (SAG1/SEN1, SAG20, SAG21, SAG101), and two at bolting (SAG12, SAG13); one gene, SAG29/SWEET15, was downregulated in 2 weeks-old plants (Figure 6E).

### 2.5. Expression of miR169H/I/J/K/L/M/N Is Downregulated in cid3cid4

**Figure 7:**
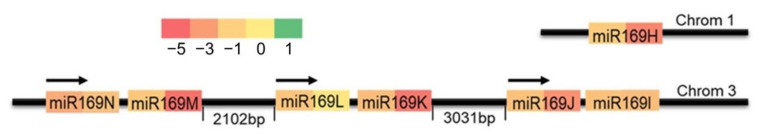
Differentially expressed subgroup miR169H/I/J/K/L/M/N in *cid3cid4*. Relative location of genes coding miR169H in *A. thaliana* chromosome 1 and miR169I/J, miR169K/L, and miR169M/N in chromosome 3; the direction of transcription is depicted by arrows. Differential expressed values are presented as described in Figure 4. Gene code values of the differentially expressed genes are displayed in Supplementary Table S1.

Further inspection of the transcriptome profile data revealed several miR169 members downregulated in the *cid3cid4* mutant. This is a large family that contains 14 members in *A. thaliana*. The downregulated genes belong to the phylogenetically related subgroup miR169H/I/J/K/L/M/N, which comprise three tandem gene pairs arranged in chromosome 3 (miR169I/J, miR169K/L, and miR169M/N) and a single gene in chromosome 1 (miR169H) [81]. With the exception of miR169N, whose expression was not affected in *cid3cid4*, all the subgroup members were downregulated (Figure 7). The genes for miR169H, miR169I, miR169J, miR169K, and miR169M were downregulated at bolting whilst miR169N was downregulated in the 2 weeks-old plants. The fact that several miR169 genes were differentially regulated in the *cid3cid4* mutant suggests an important role of the ATXN2 orthologs in the regulation of these miRNAs.

## 3. Discussion

ATXN2 is an RBP that operates in the translation regulation of specific mRNA targets across eukaryotic organisms. Posttranscriptional regulation functions of ATXN2 orthologs in several model organisms including *Saccharomyces cerevisiae*, *Caenorhabditis elegans*, *Drosophila melanogaster*, *Mus musculus*, and *Homo sapiens* have been described; indicating conserved mechanisms for ATXN2 function across eukaryotes [8]. The present study examined the phenotypic analysis of the *A. thaliana* ATXN2 orthologs *CID3* and *CID4* based on the transcriptome profiling experiments. We found that *CID3* and *CID4* may have a role mediating flowering time and a role in leaf dynamics. Both are complex biological processes that involve many components that lead to several multifaceted networks. Flowering is a developmental process that is fundamental for the continued survival of plants, dictating flowers, fruits, and seeds production. The leaf is an essential organ involved in central processes in plants, such as photosynthesis and transpiration. We assumed that the detection of differentially expressed genes involved in the flowering time and leaf dynamics pathways could shed some light on the role of the ATXN2 orthologs in plants.

During the course of evolution, plant genomes commonly experienced gene duplications events, including whole-genome duplications and small-scale duplications [82]. *A. thaliana* belongs to the Brassicaceae family, which has suffered at least three whole-genome duplication events [83]. The fact that single insertional mutants in *CID3* or *CID4* do not exhibit major macroscopic phenotypic defects, and the double *cid3cid4* mutant does, indicates that they are duplicated genes coding proteins with very similar functions (Figure 1A). Pairs of duplicated blocks that show regions of similar sequences have been uncovered in different chromosomes. In fact, *CID3* and *CID4* are within block C and block F, respectively, that showed syntenic regions [84,85]. Functional redundancy is a frequent feature among duplicated genes, though important signal transduction pathways often preserve functional copies of critical components. Moreover, redundancy does not always grant full functional overlap [86]. CID16 and CID17 comprise a subclass of plant ATXN2 orthologs lacking LsmAD. Although functional analysis of these two genes analysis is lacking, it is possible to assume that the association of ATXN2 subclasses within PABP assemblies may lead to paralog interference. Over time, retained paralogs may enhance molecular diversity and neofunctionalization [30,87,88]. These observations strengthen the notion that plants, as sessile organisms, have evolved complex mechanisms to regulate growth and development [89].

The fact that *CID3* and *CID4* may have a role mediating flowering time is supported by the fact that the floral integrator genes *FT* and *SOC1*, as well as the floral meristem identity genes *AP1*, *AP3*, *PI*, and *SEP* genes, were downregulated in the *cid3cid4* mutant, and that *FLC* the key flowering time repressor was upregulated. In the absence of *CID3* and *CID4*, promotion of flowering was delayed, and an increase of the floral repressor *FLC* was detected. It is reasonable to speculate that one or both the floral integrator genes might be direct targets of *CID3CID4* mRNAs since both were downregulated in *cid3cid4.* In the wild-type, CID3 and CID4 exert a stabilizing role on the corresponding mRNAs and therefore increase protein expression. An indirect effect on the FLC repressor may also be plausible. It has been shown that the NF-YA transcription factor represses the expression of FLC, and that NF-YA is a target of the miR169 family. Repression of *FLC* enables the expression of FT to support flowering. Yet, these conclusions were reported for miR169d, which was not differentially expressed in our conditions.

Expression of key genes in the photoperiodic flowering and the circadian clock was altered in *cid3cid4*. For instance, PRR3, PRR5, PRR7, and PRR9 regulate the transcription of CCA1 and LHY, the major oscillators in plants that activate circadian clock-regulated genes. In Drosophila, an ATXN2 ortholog has a role in the circadian clock of the nervous system, regulating the translation of the central component PERIOD1 [90,91]. Similarly, the ATXN2 ortholog in *C. elegans* is involved in the expression of LIN-42, a PERIOD ortholog that regulates germline development, and in mice, a subtle effect of the ATXN2 orthologs on circadian clock activity was inferred in an ATXN2 knockout [92,93]. Hence, a function for ATXN2 orthologs in the circadian clock might be evolutionary conserved.

Leaf development and senescence are connected processes, and several regulatory factors mediating them were differentially regulated [94]. The reduction in rosette size of *cid3cid4* suggested that leaf dynamics were affected in this double mutant. The expression of genes coding miRNA160c, miRNA164b and miRNA319 and transcription factors HB-12, NF-YA2, NF-YA10, and TCP1 was differentially regulated in *cid3cid4*. These genes are involved at distinctive points of leaf development. Except for miR164b and TCP1, which were downregulated, the other genes were upregulated in *cid3cid4*, suggesting an indirect effect of the ATXN2 orthologs. For instance, miR164 participates in leaf morphology as well as in senescence, and TCP1 participates in leaf morphology mediated by the hormone to strigolactone [59].

Leaf development ends with its senescence. Several transcription factors are involved in leaf senescence. Among them, NAC and WRKY are well known for having important roles during leaf senescence [63]. Ten NAC and eight WRKY transcription factors involved in leaf senescence were differentially expressed in *cid3cid4*. Except for WRKY22, all of them were upregulated in *cid3cid4*, suggesting that the effect of CID3 and CID4 on them is not direct. Similarly, except SAG29, which was downregulated, other differentially expressed SAG genes were upregulated in *cid3cid4*. The transition to flowering in *A. thaliana* normally concurs with the onset of senescence, and at the vegetative to reproductive stage transition, gene expression related to leaf senescence rises in mature leaves and is maintained until the flowering process ends [95,96]. In our transcriptome profiling experiments, samples at bolting were obtained at the same developmental stage in both Col-0 wild-type and *cid3cid4* mutant as soon as 10% of the plant sample began to bolt. The fact that several well-known regulators of senescence and SAG genes are upregulated in the *cid3cid4* mutant suggests that the beginning of senescence is not synchronized with the flowering time in *cid3cid4*. It is likely that a key regulator of such a process is a target of the ATXN2 orthologs.

The current findings shed some light on the role of ATXN2 orthologs in plants, providing basic information of the transcriptome dependent on *CID3* and *CID4*, two *A. thaliana* ATXN2 orthologs. Remarkably, the function of *CID3* and *CID4* in both flowering and leaf dynamics, two complex processes that involve mechanisms of post-transcriptional or translation regulation, indicate that the role of ATXN2 as a post-transcriptional regulator may have also evolved in plants. Although the targets of ATXN2 in plants are unknown, we hypothesize that ATXN2 orthologs might provide an mRNA stability mechanism to fine-tune the already complex regulation of the floral integrator FT expression. The *Drosophila* ATXN*2* ortholog is a component of miRNA pathway essential translational repression [97]. Similarly, it is tempting to speculate that the plant ATXN2 orthologs are part of the molecular machinery that influences the level of particular miRNAs (i.e., miR169), thus impacting the abundance of their targets.

## 4. Materials and Methods

### 4.1. Arabidopsis thaliana Materials and Growth Conditions

*A. thaliana* ecotype Columbia-0 and the T-DNA insertion mutants, SALK_145495 and SALK_026330 disrupting *CID3* and *CID4*, respectively, were used [34]; they were acquired from Arabidopsis Biological Resource Center (ABRC at https://abrc.osu.edu/, accessed on 10 February 2014). *A*. *thaliana* seeds were germinated in MS agar medium after stratification, then transplanted to soil and allowed to grow on a chamber (Pervival AR-36L2, from Percival; Perry, Iowa, USA) set to 16-h photoperiod (8-h dark) at 23 °C. The double homozygous *cid3cid4* mutant was obtained by crossing *cid3* and *cid4* single mutants. The genotyped double homozygous *cid3cid4* line was backcrossed twice before analysis. Instructions from The SIGnAL iSect Toolbox (http://signal.salk.edu/isects.html, accessed on 10 February 2015) were followed for the polymerase chain reaction genotyping. We used a primer LBb1.3 directed to the left border of the T-DNA 5′-ATTTTGCCGATTTCGGAAC-3′, and gene-specific primers for *CID3* (LP1, 5′-TACTCGCCAGCTTATGTCCGA-3′ and RP1, 5′-GGTGCATCTTCATTGAGGTGG-3′) and for *CID4* (LP2, 5′-TCGAACAACATGTCAAATGCG-3′ and RP2, 5′-GCAGAAACGGATCAGCTGAGAG-3′). For expression analysis by RT-PCR, samples of total RNA from 2 weeks-old rosettes were isolated using the DNeasy Plant Mini Kit (QIAGEN-Mexico; Mexico City, Mexico) and samples were DNAseI treated. Reactions were performed with the Super Script One-Step RT-PCR system with Platinum Taq polymerase (Invitrogen/Thermo Fisher Scientific- Mexico; Mexico City, Mexico) using 100 ng RNA from each sample. Amplification products were fractionated into a 1.0% agarose gel. TUB2 was used as constitutive control.

### 4.2. Transcriptome Profiling

Tissue samples from the Col-0 wild type and *cid3cid4* double mutant were collected from two conditions: 2 weeks-old seedlings, and plants at the onset of bolting, as soon as 10% of the plant sample began to bolt; samples were obtained from whole rosettes (leaves and shoot apex) without root tissue. Here onwards, we denoted young plants as 2 weeks-old plants and plants at the onset of bolting as plants at bolting. Plantlets were frozen in liquid nitrogen, and RNA was processed using a RNeasy plant mini kit (QIAGEN Mexico city, Mexico). Two biological replicas were collected from groups of plants grown under similar environmental conditions. Processing of total RNA samples and microarray data analysis and normalization was done by Oaklabs GmbH (Hennigsdorf, Germany), using an ArrayXS Arabidopsis v2 (XS-5010) microarray in the Agilent 8 × 60K format including 30,541 genes. TAIR10 and/or Araport111 were accessed for gene annotation. Commonly, genes with value of log2 fold change greater than 1.7 and *p*-value less than 0.1 (calculated by two-tailed Student’s *t*-test) were referred to as differentially expressed.

To evaluate whether the differentially expressed NAC and WRKY transcription factors were overrepresented, parametrical and nonparametrical statistical test analyses were performed. Values were below 0.01 for both parametrical *t*-test and nonparametrical Wilcoxon test (grouped = 0.0028, Satterthwaite = 0.0029 and Cochran = 0.0034; Pr>│Z│= 0.0078, respectively), suggesting that differences between the number of *NAC* and *WRKY* genes in *cid3cid4* and Col-0 have a high statistical significance.

## Data Availability

The data presented in this study are available within the article or Supplementary Material.

## Supplementary Materials

Supplementary Materials can be found at https://www.mdpi.com/1422-0067/22/6/3068/s1. Figure S1: Principal component analysis (PCA) plots for 2 weeks-old and at bolting transcriptomic data. Figure S2: Gene ontology (GO) enrichment analysis of *cid3cid4* vs Col-0 microarray data. Table S1: Differentially expressed profiles of genes regulating flowering time.

## References

[1] Van Nostrand E.L., Freese P., Pratt G.A., Wang X., Wei X., Xiao R., Blue S.M., Chen J.-Y., Cody N.A., Dominguez D. (2020). A large-scale binding and functional map of human RNA-binding proteins. Nature.

[2] Dedow L.K., Bailey-Serres J. (2019). Searching for a Match: Structure, Function and Application of Sequence-Specific RNA-Binding Proteins. Plant Cell Physiol..

[3] Smith R.W., Blee T.K., Gray N.K. (2014). Poly(A)-binding proteins are required for diverse biological processes in metazoans. Biochem. Soc. Trans..

[4] Eliseeva I.A., Lyabin D.N., Ovchinnikov L.P. (2013). Poly(A)-binding proteins: Structure, domain organization, and activity regulation. Biochem. (Moscow).

[5] Kozlov G., Ménade M., Rosenauer A., Nguyen L., Gehring K. (2010). Molecular Determinants of PAM2 Recognition by the MLLE Domain of Poly(A)-Binding Protein. J. Mol. Biol..

[6] Imbert G., Saudou F., Yvert G., Devys D., Trottier Y., Garnier J.-M., Weber C., Mandel J.-L., Cancel G., Abbas N. (1996). Cloning of the gene for spinocerebellar ataxia 2 reveals a locus with high sensitivity to expanded CAG/glutamine repeats. Nat. Genet..

[7] Elden A.C., Kim H.-J., Hart M.P., Chen-Plotkin A.S., Johnson B.S., Fang X., Armakola M., Geser F., Greene R., Lu M.M. (2010). Ataxin-2 intermediate-length polyglutamine expansions are associated with increased risk for ALS. Nat. Cell Biol..

[8] Ostrowski L.A., Hall A.C., Mekhail K. (2017). Ataxin-2: From RNA Control to Human Health and Disease. Genes.

[9] Salvi J.S., Chan J.N., Szafranski K., Liu T.T., Wu J.D., Olsen J.B., Khanam N., Poon B.P., Emili A., Mekhail K. (2014). Roles for Pbp1 and Caloric Restriction in Genome and Lifespan Maintenance via Suppression of RNA-DNA Hybrids. Dev. Cell.

[10] Stubenvoll M.D., Medley J.C., Irwin M., Song M.H. (2016). ATX-2, the C. elegans ortholog of human ataxin-2, regulates centrosome size and microtubule dynamics. PLoS Genetics.

[11] Lee J., Yoo E., Lee H., Park K., Hur J.-H., Lim C. (2017). LSM12 and ME31B/DDX6 Define Distinct Modes of Posttranscriptional Regulation by ATAXIN-2 Protein Complex in Drosophila Circadian Pacemaker Neurons. Mol. Cell.

[12] Mangus D.A., Amrani N., Jacobson A. (1998). Pbp1p, a Factor Interacting withSaccharomyces cerevisiae Poly(A)-Binding Protein, Regulates Polyadenylation. Mol. Cell. Biol..

[13] Yokoshi M., Li Q., Yamamoto M., Okada H., Suzuki Y., Kawahara Y. (2014). Direct Binding of Ataxin-2 to Distinct Elements in 3′ UTRs Promotes mRNA Stability and Protein Expression. Mol. Cell.

[14] Inagaki H., Hosoda N., Tsuiji H., Hoshino S.-I. (2020). Direct evidence that Ataxin-2 is a translational activator mediating cytoplasmic polyadenylation. J. Biol. Chem..

[15] Decker C.J., Parker R. (2012). P-Bodies and Stress Granules: Possible Roles in the Control of Translation and mRNA Degradation. Cold Spring Harb. Perspect. Biol..

[16] Formicola N., Vijayakumar J., Besse F. (2019). Neuronal ribonucleoprotein granules: Dynamic sensors of localized signals. Traffic.

[17] Ostareck D.H., Vries I.S.N.-D., Ostareck-Lederer A. (2014). DDX6 and its orthologs as modulators of cellular and viral RNA expression. Wiley Interdiscip. Rev. RNA.

[18] Swisher K.D., Parker R. (2010). Localization to, and Effects of Pbp1, Pbp4, Lsm12, Dhh1, and Pab1 on Stress Granules in Saccharomyces cerevisiae. PLoS ONE.

[19] Boag P.R., Atalay A., Robida S., Reinke V., Blackwell T.K. (2008). Protection of specific maternal messenger RNAs by the P body protein CGH-1 (Dhh1/RCK) during Caenorhabditis elegans oogenesis. J. Cell Biol..

[20] Bakthavachalu B., Huelsmeier J., Sudhakaran I.P., Hillebrand J., Singh A., Petrauskas A., Thiagarajan D., Sankaranarayanan M., Mizoue L., Anderson E.N. (2018). RNP-Granule Assembly via Ataxin-2 Disordered Domains Is Required for Long-Term Memory and Neurodegeneration. Neuron.

[21] Nonhoff U., Ralser M., Welzel F., Piccini I., Balzereit D., Yaspo M.-L., Lehrach H., Krobitsch S. (2007). Ataxin-2 Interacts with the DEAD/H-Box RNA Helicase DDX6 and Interferes with P-Bodies and Stress Granules. Mol. Biol. Cell.

[22] Becker L.A., Gitler A.D. (2018). Ataxin-2 is Droppin’some knowledge. Neuron.

[23] Van Der Lee R., Buljan M., Lang B., Weatheritt R.J., Daughdrill G.W., Dunker A.K., Fuxreiter M., Gough J., Gsponer J., Jones D.T.W. (2014). Classification of Intrinsically Disordered Regions and Proteins. Chem. Rev..

[24] Eltschinger S., Loewith R., Information P.E.K.F.C. (2016). TOR Complexes and the Maintenance of Cellular Homeostasis. Trends Cell Biol..

[25] Yang Y.-S., Kato M., Wu X., Litsios A., Sutter B.M., Wang Y., Hsu C.-H., Wood N.E., Lemoff A., Mirzaei H. (2019). Yeast Ataxin-2 Forms an Intracellular Condensate Required for the Inhibition of TORC1 Signaling during Respiratory Growth. Cell.

[26] Kato M., Yang Y.-S., Sutter B.M., Wang Y., McKnight S.L., Tu B.P. (2019). Redox State Controls Phase Separation of the Yeast Ataxin-2 Protein via Reversible Oxidation of Its Methionine-Rich Low-Complexity Domain. Cell.

[27] Bar D.Z., Charar C., Dorfman J., Yadid T., Tafforeau L., Lafontaine D.L.J., Gruenbaum Y. (2016). Cell size and fat content of dietary-restricted Caenorhabditis elegans are regulated by ATX-2, an mTOR repressor. Proc. Natl. Acad. Sci. USA.

[28] Lastres-Becker I., Nonis D., Eich F., Klinkenberg M., Gorospe M., Kötter P., Klein F.A., Kedersha N., Auburger G. (2016). Mammalian ataxin-2 modulates translation control at the pre-initiation complex via PI3K/mTOR and is induced by starvation. Biochim. Biophys. Acta Mol. Basis Dis..

[29] Bravo J., Aguilar-Henonin L., Olmedo G., Guzmán P. (2005). Four distinct classes of proteins as interaction partners of the PABC domain of *Arabidopsis thaliana* Poly(A)-binding proteins. Mol. Genet. Genom..

[30] Jiménez-López D., Bravo J., Guzmán P. (2015). Evolutionary history exposes radical diversification among classes of interaction partners of the MLLE domain of plant poly(A)-binding proteins. BMC Evol. Biol..

[31] Kariola T., Brader G., Helenius E., Li J., Heino P., Palva E.T. (2006). EARLY RESPONSIVE TO DEHYDRATION 15, a Negative Regulator of Abscisic Acid Responses in Arabidopsis. Plant Physiol..

[32] Tsumoto Y., Yoshizumi T., Kuroda H., Kawashima M., Ichikawa T., Nakazawa M., Yamamoto N., Matsui M. (2006). Light-dependent polyploidy control by a CUE protein variant in Arabidopsis. Plant Mol. Biol..

[33] Jiménez-López D., Guzmán P. (2014). Insights into the evolution and domain structure of ataxin-2 proteins across eukaryotes. BMC Res. Notes.

[34] Alonso J.M., Stepanova A.N., Leisse T.J., Kim C.J., Chen H., Shinn P., Stevenson D.K., Zimmerman J., Barajas P., Cheuk R. (2003). Genome-Wide Insertional Mutagenesis of *Arabidopsis thaliana*. Science.

[35] Madrid E., Chandler J.W., Coupland G. (2021). Gene regulatory networks controlled by FLOWERING LOCUS C that confer variation in seasonal flowering and life history. J. Exp. Bot..

[36] Bouché F., Lobet G., Tocquin P., Périlleux C. (2016). FLOR-ID: An interactive database of flowering-time gene networks in *Arabidopsis thaliana*. Nucleic Acids Res..

[37] Chen D., Yan W., Fu L.-Y., Kaufmann K. (2018). Architecture of gene regulatory networks controlling flower development in *Arabidopsis thaliana*. Nat. Commun..

[38] Wils C.R., Kaufmann K. (2017). Gene-regulatory networks controlling inflorescence and flower development in *Arabidopsis thaliana*. Biochim. Biophys. Acta Bioenerg..

[39] Sasaki E., Frommlet F., Nordborg M. (2017). The genetic architecture of the network underlying flowering time variation in *Arabidopsis thaliana*. BioRxiv.

[40] Henriques R., Wang H., Liu J., Boix M., Huang L.-F., Chua N.-H. (2017). The antiphasic regulatory module comprising CDF5 and its antisense RNA FLORE links the circadian clock to photoperiodic flowering. New Phytol..

[41] Frank A., Matiolli C.C., Viana A.J., Hearn T.J., Kusakina J., Belbin F.E., Newman D.W., Yochikawa A., Cano-Ramirez D.L., Chembath A. (2018). Circadian Entrainment in Arabidopsis by the Sugar-Responsive Transcription Factor bZIP63. Curr. Biol..

[42] Lee J., Lee I. (2010). Regulation and function of SOC1, a flowering pathway integrator. J. Exp. Bot..

[43] Abe M., Kosaka S., Shibuta M., Nagata K., Uemura T., Nakano A., Kaya H. (2019). Transient activity of the florigen complex during the floral transition in *Arabidopsis thaliana*. Development.

[44] Sundström J.F., Nakayama N., Glimelius K., Irish V.F. (2006). Direct regulation of the floral homeoticAPETALA1gene by APETALA3 and PISTILLATA in Arabidopsis. Plant J..

[45] Samach A., Klenz J.E., Kohalmi S.E., Risseeuw E., Haughn G.W., Crosby W.L. (1999). The UNUSUAL FLORAL ORGANS gene of *Arabidopsis thaliana* is an F-box protein required for normal patterning and growth in the floral meristem. Plant J..

[46] Wollmann H., Mica E., Todesco M., Long J.A., Weigel D. (2010). On reconciling the interactions between APETALA2, miR172 and AGAMOUS with the ABC model of flower development. Development.

[47] Zahn L.M., Kong H., Leebens-Mack J.H., Kim S., Soltis P.S., Landherr L.L., Soltis D.E., Depamphilis C.W., Ma H. (2005). The evolution of the SEPALLATA subfamily of MADS-box genes: A preangiosperm origin with multiple duplications throughout angiosperm history. Genetics.

[48] Johansson M., Staiger D. (2015). Time to flower: Interplay between photoperiod and the circadian clock. J. Exp. Bot..

[49] Golembeski G.S., Kinmonth-Schultz H.A., Song Y.H., Imaizumi T. (2014). Photoperiodic Flowering Regulation in *Arabidopsis thaliana*. Advances in Botanical Research.

[50] Goralogia G.S., Liu T.-K., Zhao L., Panipinto P.M., Groover E.D., Bains Y.S., Imaizumi T. (2017). CYCLING DOF FACTOR 1 represses transcription through the TOPLESS co-repressor to control photoperiodic flowering in Arabidopsis. Plant J..

[51] Farré E.M., Harmer S.L., Harmon F.G., Yanovsky M.J., Kay S.A. (2005). Overlapping and Distinct Roles of PRR7 and PRR9 in the Arabidopsis Circadian Clock. Curr. Biol..

[52] Para A., Farré E.M., Imaizumi T., Pruneda-Paz J.L., Harmon F.G., Kay S.A. (2007). PRR3 Is a Vascular Regulator of TOC1 Stability in the Arabidopsis Circadian Clock. Plant Cell.

[53] Toda Y., Kudo T., Kinoshita T., Nakamichi N. (2019). Evolutionary Insight into the Clock-Associated PRR5 Transcriptional Network of Flowering Plants. Sci. Rep..

[54] Doyle M.R., Davis S.J., Bastow R.M., McWatters H.G., Kozma-Bognár L., Nagy F., Millar A.J., Amasino R.M. (2002). The ELF4 gene controls circadian rhythms and flowering time in *Arabidopsis thaliana*. Nat. Cell Biol..

[55] Lin K., Zhao H., Gan S., Li G. (2019). Arabidopsis ELF4-like proteins EFL1 and EFL3 influence flowering time. Gene.

[56] Byrne M.E. (2005). Networks in leaf development. Curr. Opin. Plant Biol..

[57] Hur Y.-S., Um J.-H., Kim S., Kim K., Park H.-J., Lim J.-S., Kim W.-Y., Jun S.E., Yoon E.K., Lim J. (2014). *Arabidopsis thaliana* homeobox 12 (ATHB12), a homeodomain-leucine zipper protein, regulates leaf growth by promoting cell expansion and endoreduplication. New Phytol..

[58] Zhang M., Hu X., Zhu M., Xu M., Wang L. (2017). Transcription factors NF-YA2 and NF-YA10 regulate leaf growth via auxin signaling in Arabidopsis. Sci. Rep..

[59] Wang L., Wang B., Yu H., Guo H., Lin T., Kou L., Wang A., Shao N., Ma H., Xiong G. (2020). Transcriptional regulation of strigolactone signalling in Arabidopsis. Nat. Cell Biol..

[60] Yang T., Wang Y., Teotia S., Wang Z., Shi C., Sun H., Gu Y., Zhang Z., Tang G. (2019). The interaction between miR160 and miR165/166 in the control of leaf development and drought tolerance in Arabidopsis. Sci. Rep..

[61] Nikovics K., Blein T., Peaucelle A., Ishida T., Morin H., Aida M., Laufs P. (2006). The Balance between the MIR164A and CUC2 Genes Controls Leaf Margin Serration in Arabidopsis. Plant Cell.

[62] Bresso E.G., Chorostecki U., Rodriguez R.E., Palatnik J.F., Schommer C. (2018). Spatial Control of Gene Expression by miR319-Regulated TCP Transcription Factors in Leaf Development. Plant Physiol..

[63] Li Z., Woo H.R., Guo H. (2017). Genetic redundancy of senescence-associated transcription factors in Arabidopsis. J. Exp. Bot..

[64] Ooka H., Satoh K., Doi K., Nagata T., Otomo Y., Murakami K., Matsubara K., Osato N., Kawai J., Carninci P. (2003). Comprehensive Analysis of NAC Family Genes in Oryza sativa and *Arabidopsis thaliana*. DNA Res..

[65] Rushton P.J., Somssich I.E., Ringler P., Shen Q.J. (2010). WRKY transcription factors. Trends Plant Sci..

[66] Kim H., Kim H.J., Vu Q.T., Jung S., McClung C.R., Hong S., Nam H.G. (2018). Circadian control of ORE1 by PRR9 positively regulates leaf senescence in Arabidopsis. Proc. Natl. Acad. Sci. USA.

[67] Podzimska-Sroka D., O’Shea C., Gregersen P.L., Skriver K. (2015). NAC Transcription Factors in Senescence: From Molecular Structure to Function in Crops. Plants.

[68] Kim H.J., Park J.-H., Kim J., Kim J.J., Hong S., Kim J., Kim J.H., Woo H.R., Hyeon C., Lim P.O. (2018). Time-evolving genetic networks reveal a NAC troika that negatively regulates leaf senescence in Arabidopsis. Proc. Natl. Acad. Sci. USA.

[69] Balazadeh S., Kwasniewski M., Caldana C., Mehrnia M., Zanor M.I., Xue G.-P., Mueller-Roeber B. (2011). ORS1, an H_2_O_2_-Responsive NAC Transcription Factor, Controls Senescence in *Arabidopsis thaliana*. Mol. Plant.

[70] Wu A., Allu A.D., Garapati P., Siddiqui H., Dortay H., Zanor M.-I., Asensi-Fabado M.A., Munné-Bosch S., Antonio C., Tohge T. (2012). JUNGBRUNNEN1, a Reactive Oxygen Species–Responsive NAC Transcription Factor, Regulates Longevity in Arabidopsis. Plant Cell.

[71] Zentgraf U., Doll J. (2019). Arabidopsis WRKY53, a Node of Multi-Layer Regulation in the Network of Senescence. Plants.

[72] Doll J., Muth M., Riester L., Nebel S., Bresson J., Lee H.-C., Zentgraf U. (2020). *Arabidopsis thaliana* WRKY25 Transcription Factor Mediates Oxidative Stress Tolerance and Regulates Senescence in a Redox-Dependent Manner. Front. Plant Sci..

[73] Zhou X., Jiang Y., Yu D. (2011). WRKY22 transcription factor mediates dark-induced leaf senescence in Arabidopsis. Mol. Cells.

[74] Guo P., Li Z., Huang P., Li B., Fang S., Chu J., Guo H. (2017). A Tripartite Amplification Loop Involving the Transcription Factor WRKY75, Salicylic Acid, and Reactive Oxygen Species Accelerates Leaf Senescence. Plant Cell.

[75] Wang Y., Cui X., Yang B., Xu S., Wei X., Zhao P., Niu F., Sun M., Wang C., Cheng H. (2020). WRKY55 transcription factor positively regulates leaf senescence and the defense response by modulating the transcription of genes implicated in the biosynthesis of reactive oxygen species and salicylic acid in Arabidopsis. Development.

[76] Zhang Y., Liu Z., Wang X., Wang J., Fan K., Li Z., Lin W. (2018). DELLA proteins negatively regulate dark-induced senescence and chlorophyll degradation in Arabidopsis through interaction with the transcription factor WRKY6. Plant Cell Rep..

[77] Chen L., Xiang S., Chen Y., Ligang C., Shengyuan X. (2017). Arabidopsis WRKY45 Interacts with the DELLA Protein RGL1 to Positively Regulate Age-Triggered Leaf Senescence. Mol. Plant.

[78] Tian T., Ma L., Liu Y., Xu D., Chen Q., Li G. (2020). Arabidopsis FAR-RED ELONGATED HYPOCOTYL3 Integrates Age and Light Signals to Negatively Regulate Leaf Senescence. Plant Cell.

[79] Gepstein S., Sabehi G., Carp M.-J., Hajouj T., Nesher M.F.O., Yariv I., Dor C., Bassani M. (2003). Large-scale identification of leaf senescence-associated genes. Plant J..

[80] Li Z., Peng J., Wen X., Guo H. (2012). Gene Network Analysis and Functional Studies of Senescence-associated Genes Reveal Novel Regulators of Arabidopsis Leaf SenescenceF. J. Integr. Plant Biol..

[81] Li Y., Fu Y., Ji L., Wu C., Zheng C. (2010). Characterization and expression analysis of the Arabidopsis mir169 family. Plant Sci..

[82] Maere S., De Bodt S., Raes J., Casneuf T., Van Montagu M., Kuiper M., Van De Peer Y. (2005). Modeling gene and genome duplications in eukaryotes. Proc. Natl. Acad. Sci. USA.

[83] Kagale S., Robinson S.J., Nixon J., Xiao R., Huebert T., Condie J., Kessler D., Clarke W.E., Edger P.P., Links M.G. (2014). Polyploid Evolution of the Brassicaceae during the Cenozoic Era. Plant Cell.

[84] Blanc G., Barakat A., Guyot R., Cooke R., Delseny M. (2000). Extensive duplication and reshuffling in the Arabidopsis genome. Plant Cell.

[85] Schranz M.E., Lysak M.A., Mitchell-Olds T. (2006). The ABC’s of comparative genomics in the Brassicaceae: Building blocks of crucifer genomes. Trends Plant Sci..

[86] Kafri R., Springer M., Pilpel Y. (2009). Genetic Redundancy: New Tricks for Old Genes. Cell.

[87] Cheng F., Wu J., Cai X., Liang J., Freeling M., Wang X. (2018). Gene retention, fractionation and subgenome differences in polyploid plants. Nat. Plants.

[88] Baker C.R., Hanson-Smith V., Johnson A.D. (2013). Following Gene Duplication, Paralog Interference Constrains Transcriptional Circuit Evolution. Science.

[89] Lachowiec J., Queitsch C., Kliebenstein D.J. (2016). Molecular mechanisms governing differential robustness of development and environmental responses in plants. Ann. Bot..

[90] Lim C., Allada R. (2013). ATAXIN-2 Activates PERIOD Translation to Sustain Circadian Rhythms in Drosophila. Science.

[91] Zhang Y., Ling J., Yuan C., Dubruille R., Emery P. (2013). A Role for Drosophila ATX2 in Activation of PER Translation and Circadian Behavior. Science.

[92] Berardi S., McFall A., Toledo-Hernandez A., Coote C., Graham H., Stine L., Rhodehouse K., Auernhamer A., Van Wynsberghe P.M. (2018). The Period protein homolog LIN-42 regulates germline development in C. elegans. Mech. Dev..

[93] Pfeffer M., Gispert S., Auburger G., Wicht H., Korf H.-W. (2016). Impact of Ataxin-2 knock out on circadian locomotor behavior and PER immunoreaction in the SCN of mice. Chronobiol. Int..

[94] Koyama T. (2018). A hidden link between leaf development and senescence. Plant Sci..

[95] Miryeganeh M., Yamaguchi M., Kudoh H. (2018). Synchronisation of Arabidopsis flowering time and whole-plant senescence in seasonal environments. Sci. Rep..

[96] Hinckley W.E., Brusslan J.A. (2020). Gene expression changes occurring at bolting time are associated with leaf senescence in Arabidopsis. Plant Direct.

[97] McCann C., Holohan E.E., Das S., Dervan A., Larkin A., Lee J.A., Rodrigues V., Parker R., Ramaswami M. (2011). The Ataxin-2 protein is required for microRNA function and synapse-specific long-term olfactory habituation. Proc. Natl. Acad. Sci. USA.

