# Supplementary material for: The ATXN2 Orthologs CID3 and CID4, Act Redundantly to In-Fluence Developmental Pathways throughout the Life Cycle of Arabidopsis thaliana
Source: Int J Mol Sci. 2021 Mar 17;22(6):3068. doi: 10.3390/ijms22063068 (PMC8002431; doi:10.3390/ijms22063068)
Supplement: Supplementary file 1 [file ijms-22-03068-s001.pdf]

Supplementary Materials

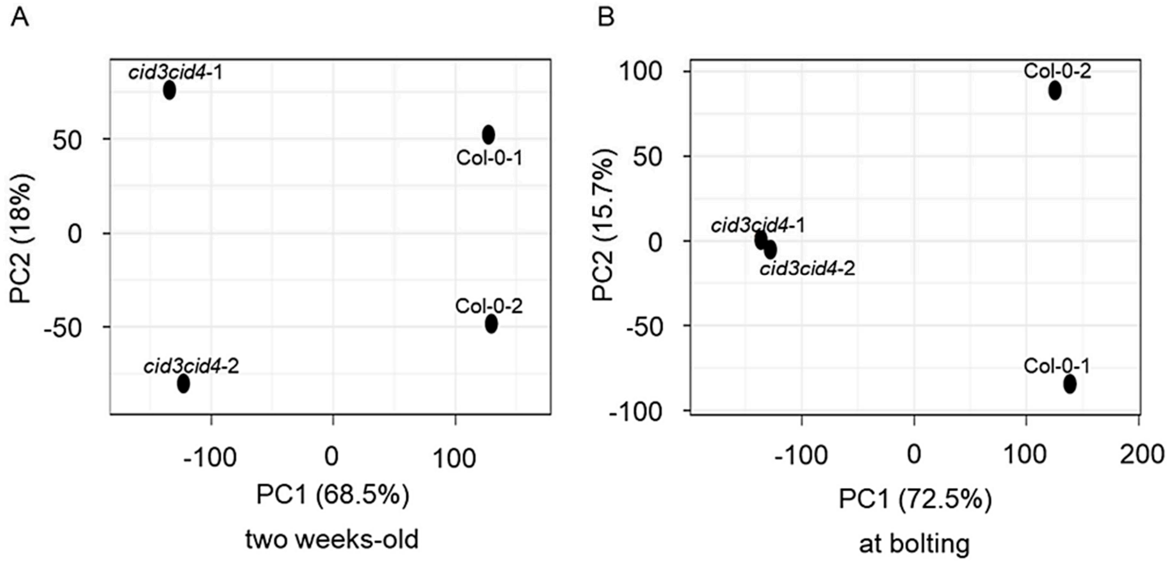

Figure S1. Principal Component Analysis (PCA) plots for two weeks-old and at bolting transcriptomic data. PCA were constructed from ClustVis [1] with Singular Value Decomposition (SVD), no transformation criteria. SVD with imputation was used to calculate principal components. X and Y represent principal component 1 (PC1) and principal component 2 (PC2), respectively, with a certain percentage of the total variance analyzed.

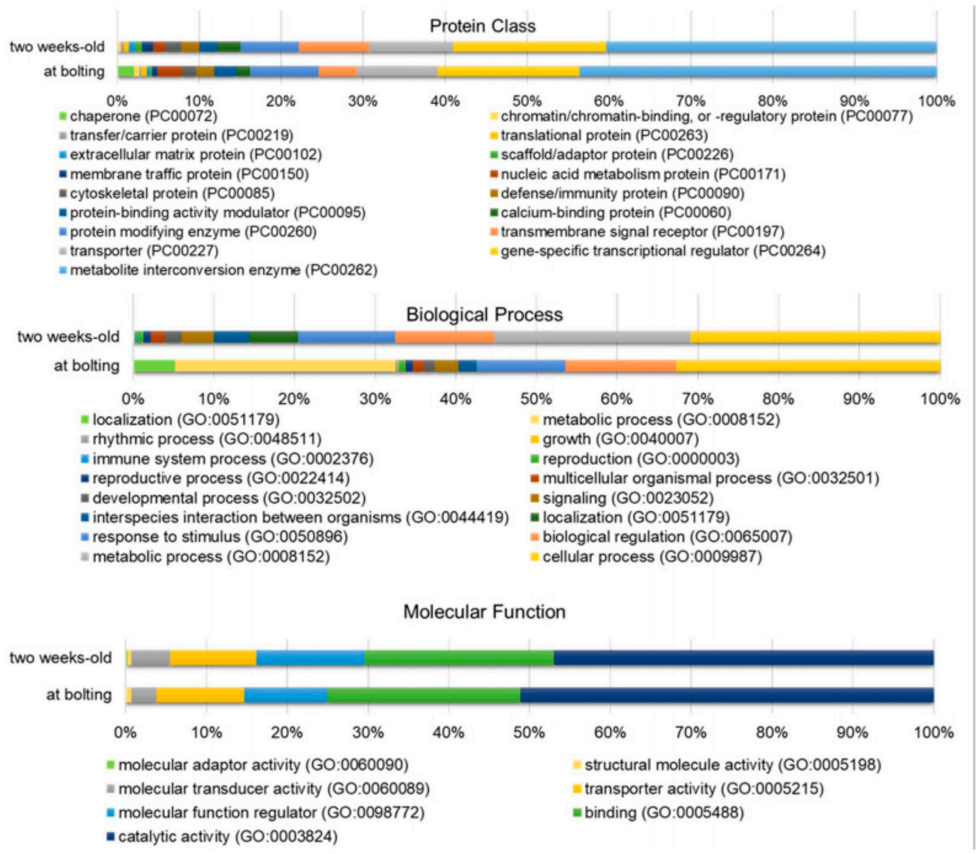

Figure S2. Gene ontology (GO) enrichment analysis of cid3cid4 vs Col-0 microarray data. GO classifications are based on PANTHER Classification System [2].

Table S1

Differentially expressed profiles of genes regulating flowering time

Experiment	TargetName	Gene Symbol	3X4-1	3X4-2	WT-1	WT-2	log2FC	p-value	Description
Two weeks	AT1G65480.1	FT	7.14611	4.91756	61.1914	64.9679	-3.410905696	0.001473818	FLOWERING LOCUS T
at Bolting	AT1G65480.1	FT	15.9146	14.7978	100.315	107.675	-2.759671629	0.001758681	FLOWERING LOCUS T
Two weeks	AT2G45660.1	SOC1	837.16	809.171	2561.61	2462.17	-1.609444707	0.000934215	AGAMOUS-like 20
at Bolting	AT2G45660.1	SOC1	2383.36	2296	2534.21	2372.55	-0.067927523	0.341470172	AGAMOUS-like 20
Two weeks	AT5G10140.1	FLC	1059.14	1022.94	128.451	119.505	3.070590958	0.000413081	Flowering Locus C
at Bolting	AT5G10140.1	FLC	439.909	474.58	72.6524	73.7458	2.642070633	0.002033355	Flowering Locus C
Two weeks	AT4G35900.1	FD	103.159	17.9955	2.46234	5.89506	3.499376843	0.316724914	BZIP14
at Bolting	AT4G35900.1	FD	9.31928	2.44579	7.1145	8.20658	-0.678512227	0.660205902	BZIP14
Two weeks	AT1G30950.1	UFO	2.93135	2.39794	13.2538	8.67829	-2.016192076	0.069096741	F-box family protein
at Bolting	AT1G30950.1	UFO	6.40816	7.58554	3.87626	3.75375	0.87008207	0.032903067	F-box family protein
Two weeks	AT4G11880.1	XAL2	18.3393	17.1485	6.46117	7.90087	1.3115351	0.007730967	AGAMOUS-like 14
at Bolting	AT4G11880.1	XAL2	22.3263	24.05	4.79328	3.96933	2.40936427	0.002569973	AGAMOUS-like 14
Two weeks	AT1G69120.1	AP1	2.46186	2.40836	36.685	34.4649	-3.868187261	0.001120742	Apetala 1
at Bolting	AT1G69120.1	AP1	83.3457	79.9078	121.898	122.851	-0.584498071	0.001910843	Apetala 1
Two weeks	AT3G54340.1	AP3	2.46234	2.51359	66.9726	65.1255	-4.730435191	0.00021122	Apetala 3
at Bolting	AT3G54340.1	AP3	306.506	273.989	371.672	384.09	-0.38272056	0.037250902	Apetala 3
Two weeks	AT5G20240.1	PI	47.413	42.1952	190.419	170.992	-2.012300442	0.005432554	PISTILLATA
at Bolting	AT5G20240.1	PI	1069.37	1058.97	1295.63	1340.82	-0.308673262	0.008226177	PISTILLATA
Two weeks	AT1G26310.1	CAL	2.35066	5.08469	11.6813	13.1178	-1.840178556	0.030210373	CAULIFLOWER
at Bolting	AT1G26310.1	CAL	26.6122	22.253	32.1497	33.5962	-0.433486385	0.066705225	CAULIFLOWER
Two weeks	AT5G15800.2	SEP1,	3.92464	4.11416	54.1306	52.5451	-3.730349289	0.000261966	SEPALLATA1
at Bolting	AT5G15800.2	SEP1,	234.258	217.816	542.48	560.039	-1.286944498	0.001364931	SEPALLATA1
Two weeks	AT3G02310.1	SEP2,	2.72646	7.16261	28.4525	34.5913	-2.827653232	0.019704782	SEPALLATA2
at Bolting	AT3G02310.1	SEP2,	151.844	157.346	210.714	218.478	-0.473121889	0.006229407	SEPALLATA2
Two weeks	AT1G24260.2	SEP3,	14.2965	15.3006	41.5416	46.1284	-1.565480906	0.006473932	SEPALLATA3
at Bolting	AT1G24260.2	SEP3,	175.476	174.017	302.845	311.459	-0.813559938	0.001086708	SEPALLATA3
Two weeks	AT2G03710.1	SEP4,	34.6352	33.2337	90.4303	100.869	-1.493165639	0.007202701	SEPALLATA4
at Bolting	AT2G03710.1	SEP4,	85.1488	80.9535	163.248	165.187	-0.983971003	0.000809593	SEPALLATA4
Two weeks	AT5G04275.1	miR172B	6.72058	7.74538	8.41801	9.697	-0.32454797	0.155882823	miR172
at Bolting	AT5G04275.1	miR172B	22.9713	25.2518	3.59481	6.57053	2.309074828	0.009565252	miR172
Two weeks	AT4G18960.1	AG	27.5358	31.99	41.4831	46.9327	-0.572093827	0.054542609	Agamous
at Bolting	AT4G18960.1	AG	104.821	101.205	116.771	124.694	-0.228432864	0.055420504	Agamous
Two weeks	AT5G62430.1	CDF1	239.803	253.061	1138.79	1051.19	-2.151023241	0.00271425	cycling DOF factor 1
at Bolting	AT5G62430.1	CDF1	936.12	1048.08	1003.97	954.425	0.017047164	0.852591934	cycling DOF factor 1
Two weeks	AT1G69570.1	CDF5	660.646	738.292	641.789	704.386	0.054801827	0.649633751	cycling DOF factor 5
at Bolting	AT1G69570.1	CDF5	1334.33	1331.94	1266.2	1295.27	0.057943222	0.069485817	cycling DOF factor 5
Two weeks	AT1G69572.1	FLORE	121.716	103.36	48.8293	45.4072	1.25219241	0.019764697	other RNA
at Bolting	AT1G69572.1	FLORE	145.452	138.047	19.9177	19.5841	2.842912484	0.000921638	other RNA
Two weeks	AT1G65480.1	FT	7.14611	4.91756	61.1914	64.9679	-3.410905696	0.001473818	FLOWERING LOCUS T
at Bolting	AT1G65480.1	FT	15.9146	14.7978	100.315	107.675	-2.759671629	0.001758681	FLOWERING LOCUS T
Two weeks	AT2G46790.1	PRR9	177.808	169.351	553.521	612.201	-1.746153356	0.005204791	pseudo-response regulator 9
at Bolting	AT2G46790.1	PRR9	321.111	334.125	198.507	201.675	0.711118941	0.002746421	pseudo-response regulator 9
Two weeks	AT5G24470.1	PRR5	46.5182	46.1284	13.9695	13.9047	1.732798762	3.7215E-05	pseudo-response regulator 5
at Bolting	AT5G24470.1	PRR5	9.46033	7.31747	6.65489	8.33457	0.159856423	0.578770882	pseudo-response regulator 5
Two weeks	AT5G60100.2	PRR3	51.1127	6.62959	2.87014	2.26456	2.852088837	0.358529217	pseudo-response regulator 3
at Bolting	AT5G60100.2	PRR3	15.2015	14.8396	2.27037	4.21628	2.279311986	0.006987099	pseudo-response regulator 3
Two weeks	AT5G02810.1	PRR7	2444.06	2737.35	3184.67	3305.8	-0.327041859	0.05404	pseudo-response regulator 7
at Bolting	AT5G02810.1	PRR7	1484.93	1397.05	1534.79	1505.81	-0.077901218	0.228633447	pseudo-response regulator 7
Two weeks	AT5G28770.2	ATBZIP63, BZO2H3	2004.58	2021	1317.28	1393.62	0.570985023	0.003509253	ATBZIP63, BZO2H3
at Bolting	AT5G28770.2	ATBZIP63, BZO2H3	471.202	488.155	2070.43	2009.27	-2.088386544	0.000413438	ATBZIP63, BZO2H3
Two weeks	AT2G40080.1	ELF4	42.9306	46.46	23.9499	68.6302	0.13956615	0.949743569	EARLY FLOWERING 4
at Bolting	AT2G40080.1	ELF4	116.318	117.89	22.3117	26.6122	2.264741841	0.000610142	EARLY FLOWERING 4
Two weeks	AT2G29950.1	EFL1	62.9919	61.5902	33.6336	31.2473	0.942116199	0.002142001	ELF4-like 1
at Bolting	AT2G29950.1	EFL1	126.676	130.468	19.03	20.0078	2.719928652	0.000322217	ELF4-like 1
Two weeks	AT2G06255.1	EFL3	332.535	309.486	209.71	198.938	0.651332904	0.011677234	ELF4-like 3
at Bolting	AT2G06255.1	EFL3	304.968	320.06	99.4721	101.358	1.637588227	0.001283067	ELF4-like 3
Two weeks	AT2G40080.1	EFL4	42.9306	46.46	23.9499	68.6302	0.13956615	0.949743569	ELF4-like 4
at Bolting	AT2G40080.1	EFL4	116.318	117.89	22.3117	26.6122	2.264741841	0.000610142	ELF4-like 4

Differentially expressed profiles of genes regulating leaf dynamics

Experiment	TargetName	Gene Symbol	3X4-1	3X4-2	WT-1	WT-2	log2FC	p-value	Description
Two weeks	AT5G41663.1	miR319	3.43549	2.30485	12.4942	9.61922	-1.961958896	0.033806123	MIR319/MIR319B; miRNA
at Bolting	AT5G41663.1	miR319	31.8695	29.8601	9.40572	2.434332	2.097449007	0.020500424	MIR319/MIR319B; miRNA
Two weeks	AT5G01747.1	miR164	5.94618	8.07917	17.7961	43.531	-2.005645119	0.208456691	MIR164/MIR164B; miRNA
at Bolting	AT5G01747.1	miR164	11.2533	9.92321	5.51098	2.2462	1.586643914	0.062603053	MIR164/MIR164B; miRNA
Two weeks	AT5G46845.1	miR160	2.47442	5.41442	2.92718	3.76482	0.140902424	0.733196987	MIR160/MIR160C (MICRORNA160); miRNA
at Bolting	AT5G46845.1	miR160	12.6167	10.7663	2.48641	2.46916	2.233814398	0.009934137	MIR160/MIR160C (MICRORNA160); miRNA
Two weeks	AT3G61890.1	HB-12	1261.91	1321.56	1461.82	1410.37	-0.152994212	0.067040413	homeobox 12
at Bolting	AT3G61890.1	HB-12	7592.06	7665.33	1349.5	1353.15	2.497046273	3.41422E-05	homeobox 12
Two weeks	AT1G67260.2	TCP1	3.2648	3.87448	63.3734	64.2492	-4.165207393	7.84364E-05	TCP family transcription factor, TCP1
at Bolting	AT1G67260.1	TCP1	2.40794	2.46556	16.076	3.52886	-1.628161984	0.361243131	TCP family transcription factor, TCP1
Two weeks	AT3G05690.1	NF-YA2	306.366	301.703	206.399	200.65	0.579134052	0.001353246	nuclear factor Y, subunit A2
at Bolting	AT3G05690.1	NF-YA2	631.988	627.32	175.139	174.887	1.847087646	2.64308E-05	nuclear factor Y, subunit A2
Two weeks	AT5G06510.1	NF-YA10	98.5012	115.24	67.4283	74.2898	0.590096297	0.057684884	nuclear factor Y, subunit A10
at Bolting	AT5G06510.1	NF-YA10	338.065	317.017	55.9449	66.7857	2.421085605	0.00197208	nuclear factor Y, subunit A10
Two weeks	AT2G40740.1	WRKY55	104.289	116.876	8.68083	14.9139	3.278429713	0.005016153	WRKY DNA-binding protein 55
at Bolting	AT2G40740.1	WRKY55	40.8288	38.0024	8.00684	5.68217	2.545929458	0.003141167	WRKY DNA-binding protein 55
Two weeks	AT1G62300.1	WRKY6	1515.93	1442.89	274.918	272.134	2.43485103	0.00091724	WRKY DNA-binding protein 6
at Bolting	AT1G62300.1	WRKY6	415.409	417.991	772.663	697.374	-0.81688377	0.013714606	WRKY DNA-binding protein 6
Two weeks	AT3G01970.1	WRKY45	170.781	173.266	101.058	112.398	0.690668081	0.00781013	WRKY DNA-binding protein 45
at Bolting	AT3G01970.1	WRKY45	354.181	371.972	40.7714	45.1024	3.081387668	0.000816832	WRKY DNA-binding protein 45
Two weeks	AT4G18170.1	WRKY28	331.816	306.641	49.7194	82.5321	2.316013008	0.006608998	WRKY DNA-binding protein 28
at Bolting	AT4G18170.1	WRKY28	61.095	63.5704	81.8404	71.3969	-0.294632575	0.11688089	WRKY DNA-binding protein 28
Two weeks	AT4G01250.1	WRKY22	1364.19	1369.78	434.622	449.645	1.628648677	7.5089E-05	WRKY DNA-binding protein 22
at Bolting	AT4G01250.1	WRKY22	222.678	217.971	975.43	939.376	-2.119322922	0.000607804	WRKY DNA-binding protein 22
Two weeks	AT4G31800.1	WRKY18	8314.22	8552.32	1059.78	1036.65	3.008101533	0.000262216	WRKY DNA-binding protein 18
at Bolting	AT4G31800.1	WRKY18	2879.3	2767.38	7418.28	7557.9	-1.40741564	0.000367674	WRKY DNA-binding protein 18
Two weeks	AT2G30250.1	WRKY25	2902.78	3034.57	658.531	649.795	2.181773763	0.00081313	WRKY DNA-binding protein 25
at Bolting	AT2G30250.1	WRKY25	831.682	831.945	592.219	652.495	0.42021127	0.020082213	WRKY DNA-binding protein 25
Two weeks	AT4G23810.1	WRKY53	11516.7	11579	2712.87	2782.95	2.071329667	2.8384E-05	WRKY DNA-binding protein 53
at Bolting	AT4G23810.1	WRKY53	2973.61	2944.24	5245.77	5218.63	-0.822357475	7.73539E-05	WRKY DNA-binding protein 53
Two weeks	AT5G13080.1	WRKY75	352.439	348.518	43.5925	42.8994	3.018712762	4.19889E-05	WRKY DNA-binding protein 75
at Bolting	AT5G13080.1	WRKY75	263.576	271.818	2.23703	2.25231	6.897791187	0.000240922	WRKY DNA-binding protein 75
Two weeks	AT3G29035.1	ORS1	92.3481	87.3304	45.5492	47.3821	0.950899185	0.003770796	NAC domain containing protein 3, ORE1 SISTER1, ORS1

Table S1. *Cont.*

at Bolting	AT3G29035.1	ORS1	166.17	167.87	33.6213	30.9606	2.372025812	0.000137276	NAC domain containing protein 3, ORE1 SISTER1, ORS1
Two weeks	AT2G43000.1	JUB1	203.211	202.515	32.1007	29.541	2.719770019	5.94274E-05	NAC domain containing protein 42, JUB1, JUNGBRUNNEN 1,
at Bolting	AT2G43000.1	JUB1	125.944	108.796	10.1393	15.4164	3.226920067	0.007276203	NAC domain containing protein 42, JUB1, JUNGBRUNNEN 1,
Two weeks	AT5G39610.1	ORE1	848.797	860.683	519.275	542.089	0.687936514	0.001571692	NAC domain containing protein 6, ORESARA1 (ORE1)
at Bolting	AT5G39610.1	ORE1	1714.56	1784.17	132.556	136.814	3.699064913	0.000466046	NAC domain containing protein 6, ORESARA1 (ORE1)
Two weeks	AT1G69490.1	NAC029, AtNAP	1383.88	1386.11	1250.46	1229.99	0.159327553	0.005019527	NAC domain containing protein 29, NAC-like, activated by AP3/PI
at Bolting	AT1G69490.1	NAC029, AtNAP	3911.36	3893.28	960.318	967.475	2.017388011	1.09477E-05	NAC domain containing protein 29, NAC-like, activated by AP3/PI
Two weeks	AT5G18270.1	NAC087	392.073	418.782	76.9247	90.7425	2.277976299	0.002178797	NAC domain containing protein 87
at Bolting	AT5G18270.1	NAC087	352.845	351.28	202.145	206.463	0.785191113	0.000241459	NAC domain containing protein 87
Two weeks	AT5G22380.1	NAC090	458.353	458.353	33.7137	33.7667	3.763919131	3.89498E-09	NAC domain containing protein 90
at Bolting	AT5G22380.1	NAC090	52.1696	59.0766	40.9124	50.2162	0.292552569	0.224668951	NAC domain containing protein 90
Two weeks	AT1G34180.1	NAC016	9.84378	9.69542	2.94822	3.27256	1.653125992	0.000716373	NAC domain containing protein 16
at Bolting	AT1G34180.1	NAC016	4.22986	7.36779	2.81972	2.46394	1.082661411	0.183593065	NAC domain containing protein 16
Two weeks	AT4G27410.2	NAC072	1429.43	1407.19	1386.11	1502.14	-0.024901962	0.704758363	NAC domain containing protein 72
at Bolting	AT4G27410.2	NAC072	2019.53	2158.7	687.036	683.98	1.606849346	0.002449917	NAC domain containing protein 72
Two weeks	AT1G52890.1	NAC019	749.794	746.322	460.489	429.069	0.750958065	0.00270504	NAC domain containing protein 19
at Bolting	AT1G52890.1	NAC019	547.345	530.122	64.8209	75.1974	2.947744622	0.000459741	NAC domain containing protein 19
Two weeks	AT3G15500.1	NAC055	149.071	146.561	64.6541	51.3247	1.359480959	0.005651844	NAC domain containing protein 055
at Bolting	AT3G15500.1	NAC055	79.3135	82.8511	12.6249	15.4016	2.539367553	0.001122137	NAC domain containing protein 055
Two weeks	AT2G46790.1	PRR9	177.808	169.351	553.521	612.201	-1.746153356	0.005204791	pseudo-response regulator 9
at Bolting	AT2G46790.1	PRR9	321.111	334.125	198.507	201.675	0.711118941	0.002746421	pseudo-response regulator 9
Two weeks	AT4G25490.1	CBF1	133.889	137.896	22.7938	21.2146	2.62738765	0.000357348	C-repeat/DRE binding factor 1
at Bolting	AT4G25490.1	CBF1	20.6618	21.4297	288.412	294.851	-3.792697936	0.000143551	C-repeat/DRE binding factor 1
Two weeks	AT4G25470.1	CBF2	312.206	306.787	166.55	73.2642	1.486299973	0.05570442	C-repeat/DRE binding factor 2
at Bolting	AT4G25470.1	CBF2	68.8813	77.9768	1168.85	1228.59	-4.031324201	0.000720151	C-repeat/DRE binding factor 2
Two weeks	AT1G66390.1	MYB90	32.8376	33.8333	95.4815	87.6233	-1.456371596	0.004596235	myb domain protein 90
at Bolting	AT1G66390.1	MYB90	530.928	545.529	24.9229	22.9187	4.493015574	0.000205226	myb domain protein 90
Two weeks	AT2G47190.1	MYB2	7.79314	11.9518	5.97355	7.33683	0.543807661	0.279284732	myb domain protein 2
at Bolting	AT2G47190.1	MYB2	17.4185	17.4313	2.775	2.46264	2.736731617	0.000111436	myb domain protein 2
Two weeks	AT4G09820.1	TT8	161.46	152.277	41.6508	34.6259	2.045767631	0.002362287	basic helix-loop-helix (bHLH) DNA-binding superfamily protein
at Bolting	AT4G09820.1	TT8	41.8226	45.7673	107.006	109.706	-1.308295188	0.001367747	basic helix-loop-helix (bHLH) DNA-binding superfamily protein
Two weeks	AT2G29350.1	SAG13	2119.56	2211.17	957.546	947.21	1.184702418	0.001441012	senescence-associated gene 13
at Bolting	AT2G29350.1	SAG13	3829.37	3751.9	569.639	587.481	2.711997256	0.000153102	senescence-associated gene 13
Two weeks	AT3G10985.1	SAG20	19731.6	19867.9	5753.43	6155.67	1.734232668	0.00023516	senescence associated gene 20
at Bolting	AT3G10985.1	SAG20	10915.4	11652	14640.3	14444.6	-0.366766382	0.013400601	senescence associated gene 20
Two weeks	AT4G02380.1	SAG21, LEA38	48287.4	52178.6	7839.6	7776.41	2.68453844	0.002097057	senescence-associated gene 21
at Bolting	AT4G02380.1	SAG21, LEA38	13257.9	13752.3	19120.7	19067.8	-0.49987427	0.001972707	senescence-associated gene 21
Two weeks	AT5G13170.1	SAG29, SWEET15	12.1037	15.0032	151.414	174.603	-3.592857383	0.00605704	senescence-associated gene 29
at Bolting	AT5G13170.1	SAG29, SWEET15	378.606	403.637	791.538	793.97	-1.019993704	0.000978771	senescence-associated gene 29
Two weeks	AT5G14930.1	SAG101	149.197	159.352	40.0512	37.9506	1.98366184	0.002017069	senescence-associated gene 101
at Bolting	AT5G14930.1	SAG101	53.2594	46.1793	68.7878	60.3414	-0.377509202	0.114589669	senescence-associated gene 101
Two weeks	AT5G45890.1	SAG12	5.45877	3.37419	9.91583	12.3908	-1.368906922	0.053124162	senescence-associated gene 12
at Bolting	AT5G45890.1	SAG12	438.377	454.744	3.22624	2.87426	7.195947377	0.000340447	senescence-associated gene 12
Two weeks	AT4G35770.1	SAG1, SEN1	5614.27	5646.84	1111.91	1091.58	2.353542158	1.79676E-05	SEN1, SENESCENCE ASSOCIATED GENE 1
at Bolting	AT4G35770.1	SAG1, SEN1	1917.3	1953.26	2108.27	2021	-0.093084434	0.111320057	SEN1, SENESCENCE ASSOCIATED GENE 1

Differentially expressed profiles of miR169 genes

Experiment	TargetName	Gene Symbol	3X4-1	3X4-2	WT-1	WT-2	log2FC	p-value	Description
Two weeks	AT1G19371.1	miR169H	17.2217	14.3896	39.1221	34.0912	-1.214064573	0.018719195	MIR169H; miRNA
at Bolting	AT1G19371.1	miR169H	3.31215	3.98462	29.3909	90.962	-3.831139773	0.207760959	MIR169H; miRNA
Two weeks	AT1G53683.1	miR169D	7.6791	7.17507	11.9236	14.8276	-0.841015516	0.056248433	MIR169D; miRNA
at Bolting	AT1G53683.1	miR169D	2.50061	6.59779	8.99605	22.2867	-1.801569929	0.251741258	MIR169D; miRNA
Two weeks	AT1G53687.1	miR169E	3.78382	2.38197	3.29031	5.91284	-0.555038026	0.414496716	MIR169E; miRNA
at Bolting	AT1G53687.1	miR169E	2.33523	2.35113	2.45719	3.905	-0.402706502	0.36665096	MIR169E; miRNA
Two weeks	AT3G13405.1	miR169A	22.3117	20.02	33.5481	34.8747	-0.694584726	0.010143659	MIR169A; miRNA
at Bolting	AT3G13405.1	miR169A	10.3235	9.13312	28.6525	23.2675	-1.410932681	0.027668305	MIR169A; miRNA
Two weeks	AT3G14385.1	miR169F	9.86905	10.2579	15.2893	9.37918	-0.251168762	0.523359987	MIR169F; miRNA
at Bolting	AT3G14385.1	miR169F	7.92976	7.84553	10.6017	13.1393	-0.581446058	0.088340589	MIR169F; miRNA
Two weeks	AT3G26812.1	miR169I	4.91208	4.29826	20.4617	9.06913	-1.567865728	0.216852287	MIR169I; miRNA
at Bolting	AT3G26812.1	miR169I	2.52267	2.54812	6.92409	11.3378	-1.805154291	0.096093003	MIR169I; miRNA
Two weeks	AT3G26813.1	miR169	43.7867	38.9442	115.751	128.262	-1.561036724	0.006847952	MIR169J; miRNA
at Bolting	AT3G26813.1	miR170	9.12249	7.34047	85.9478	86.4698	-3.397106575	0.000141739	MIR169J; miRNA
Two weeks	AT3G26815.1	miR169K	15.2109	16.4394	58.6192	47.8811	-1.744283207	0.020220352	MIR169K; miRNA
at Bolting	AT3G26815.1	miR169K	2.1682	2.522	45.6342	50.8714	-4.364880156	0.003252534	MIR169K; miRNA
Two weeks	AT3G26816.1	miR169L	2.68524	2.3922	8.97999	5.58858	-1.482903272	0.108161679	MIR169L; miRNA
at Bolting	AT3G26816.1	miR169L	2.30862	2.3489	2.42388	3.03229	-0.219354997	0.320491573	MIR169L; miRNA
Two weeks	AT3G26818.1	miR169M	15.5222	20.8173	41.5267	49.485	-1.334457248	0.02923521	MIR169M; miRNA
at Bolting	AT3G26818.1	miR169M	2.20422	2.21942	73.1437	40.6894	-4.624395936	0.077853508	MIR169M; miRNA
Two weeks	AT3G26819.1	miR169N	4.34576	2.49443	18.7575	17.2217	-2.448616312	0.006745667	MIR169N; miRNA
at Bolting	AT3G26819.1	miR169N	2.58391	2.44048	8.45123	6.78491	-1.592385129	0.02579073	MIR169N; miRNA
Two weeks	AT4G21595.1	miR169G	2.47604	4.45715	2.53885	2.55907	0.382180682	0.45206366	MIR169G; miRNA
at Bolting	AT4G21595.1	miR169G	2.46161	2.49896	2.68697	2.55888	-0.080281344	0.165936442	MIR169G; miRNA
Two weeks	AT5G24825.1	miR169B	12.6249	5.383	3.52438	2.69095	1.420562706	0.247128929	MIR169B; miRNA
at Bolting	AT5G24825.1	miR169B	7.413	4.91326	2.99717	6.83111	0.415508579	0.639955459	MIR169B; miRNA
Two weeks	AT5G39635.1	miR169C	2.30011	2.29766	2.28847	2.28636	0.007216113	0.019293394	MIR169C; miRNA
at Bolting	AT5G39635.1	miR169C	2.27535	2.28365	2.29527	2.27123	-0.002353823	0.795866268	MIR169C; miRNA

References

1. Metsalu, T.; Vilo, J. ClustVis: a web tool for visualizing clustering of multivariate data using Principal Component Analysis and heatmap. *Nucleic Acids Res.* **2015**, *43*, W566–W570, doi:10.1093/nar/gkv468.
2. Mi, H.; Muruganujan, A.; Huang, X.; Ebert, D.; Mills, C.; Guo, X.; Thomas, P.D. Protocol Update for large-scale genome and gene function analysis with the PANTHER classification system (v.14.0). *Nat. Protoc.* **2019**, *14*, 703–721, doi:10.1038/s41596-019-0128-8.
